# Exposure of cells to prions induces prion strain-dependent changes in mitochondrial redox state and respiration

**DOI:** 10.1242/jcs.264762

**Published:** 2026-09-25

**Authors:** Daniel Shoup, Leah R. Varner, Brent Race, Elyssa Hawk, Suzette A. Priola

**Affiliations:** Rocky Mountain Laboratories, Laboratory of Neurological Infections and Immunity, National Institute of Allergy and Infectious Diseases, National Institutes of Health, 903 S. 4th St, Hamilton, MT 59840, USA

**Keywords:** Protein misfolding diseases, Proteinopathies, Protein aggregation, Mitochondrial dysfunction, Mitochondrial respiration, Mitochondrial redox state

## Abstract

Prion diseases are transmissible, neurodegenerative diseases caused by misfolded, protease-resistant and infectious aggregates of the mammalian prion protein (PrP^Sc^) that replicate by converting properly folded prion protein (PrP^C^, encoded by *PRNP* in humans) into PrP^Sc^. Spongiform change and cellular loss in the brain are hallmarks of prion disease, but our understanding of how prions alter cellular fitness remains incomplete. Here, we characterized changes in mitochondrial redox state and respiration in mouse neural cells following uptake of two different PrP^Sc^ strains: 22L and 87V. Only 22L PrP^Sc^ induced changes in cellular respiration and mitochondrial redox state, even in cells that did not produce PrP^C^. These effects were disrupted by treatment with detergent and dependent upon endolysosomal acidification, suggesting that both PrP^Sc^ membrane association and lysosomal degradation are involved. Interestingly, cells chronically infected with 22L appeared to adapt to infection, showing no signs of mitochondrial dysfunction, but were more susceptible to oxidative stress even though mitochondrial respiration was normal. Thus, during initial prion infection, PrP^Sc^ drives mitochondrial dysfunction in a manner that is both strain dependent and independent of PrP^C^ expression, whereas persistent prion infection increases mitochondrial sensitivity to cellular stress.

## Teaser

**Highlighted Article:** Infectious prions can drive changes in mitochondrial fitness and respiration in a strain-specific manner, which cells adapt to during long exposure.

## INTRODUCTION

Infectious prions are protease-resistant aggregates of misfolded mammalian prion protein (PrP^Sc^) that are the causative agents of lethal and transmissible neurodegenerative diseases such as Creutzfeldt–Jakob disease in humans and scrapie in sheep (Colby and Prusiner, 2011). Prion diseases can be caused by numerous different prion strains (Kraus et al., 2021; Hoyt et al., 2022) that vary in the progression and pathology of the diseases they cause (Bessen and Marsh, 1992; Morales et al., 2007) as well as in the structural properties of PrP^Sc^ (Atarashi et al., 2006; Bessen et al., 1995; Legname et al., 2006; Hoyt et al., 2022). Aggregates of PrP^Sc^ propagate through cycles of growth by seeded polymerization, wherein properly folded prion protein (PrP^C^, encoded by *PRNP* in humans) binds to and acquires the same conformation as PrP^Sc^, followed by processes that induce fragmentation of PrP^Sc^ aggregates that can increase the number of seeding particles (Tuite and Koloteva-Levin, 2004; Meisl et al., 2021; Gill and Castle, 2018).

Infectious prions primarily localize along membranes at the cell surface (Dearmond and Bajsarowicz, 2010) and throughout the endosomal trafficking network (Arnold et al., 1995; Vey et al., 1996) with the help of a glycosylphosphatidylinositol (GPI) anchor (Klingeborn et al., 2011). While GPI-anchored prions are cycled in (Fehlinger et al., 2017) and out (Fevrier et al., 2004) of cells through many different routes, prions are also trafficked to the lysosome for degradation (Sakaguchi and Uchiyama, 2017; Caughey et al., 1991). Lysosomal degradation of PrP^Sc^ is facilitated by endolysosomal acidification (Caughey et al., 1991; Shoup and Priola, 2021) as prion protein structure is destabilized at low pH (Shoup and Priola, 2021, 2024). However, infectious prions are highly protease resistant, and there is evidence from work with mouse prions that a significant proportion of PrP^Sc^ aggregates can survive lysosomal degradation (Hutti et al., 2020; Mayer-Sonnenfeld et al., 2008; Shoup and Priola, 2021).

Prion diseases are ultimately fatal, but the mechanism of lethality by infectious prion protein variants is not well understood. Although deletion of the prion protein is not lethal (Büeler et al., 1993), the sudden loss of PrP^C^ following conversion to PrP^Sc^ might disrupt cellular systems that PrP^C^ has been found to contribute to, such as those responsible for regulating metal homeostasis (Brown et al., 1997; Ashok and Singh, 2018) and redox stress (Foliaki et al., 2023; Watt et al., 2005). Alternatively, the prion protein might disrupt cellular homeostasis through a gain of function upon conversion of PrP^C^ to PrP^Sc^, allowing PrP^Sc^ to potentially interact aberrantly with proteins involved with metal homeostasis and redox stress. Other changes to cellular fitness that have been observed during prion infection, such as abnormalities in lysosomal activity (Hutti et al., 2020; Uchiyama et al., 2017) and mitochondria (Keller et al., 2019; Faris et al., 2017b), might also result from the prion protein acquiring novel function following conversion to PrP^Sc^.

How PrP^Sc^, which primarily localizes along the endolysosomal pathway (Arnold et al., 1995; Vey et al., 1996) and cell surface (Dearmond and Bajsarowicz, 2010; Caughey et al., 1991), influences cytoplasmic cellular components, such as mitochondria (Keller et al., 2019; Faris et al., 2017b), is not well understood, but altered mitochondrial respiration has been observed in prion-infected rodents (Faris et al., 2017b; Ward et al., 2022; Choi et al., 2014). There is evidence suggesting that PrP^Sc^ can enter the cytoplasm (Homma et al., 2014), possibly through lysosomal leakage as a result of PrP^Sc^ accumulation (Goold et al., 2015). As PrP^C^ has been found to localize to mitochondria (Faris et al., 2017a), conversion of PrP^C^ to PrP^Sc^ at the mitochondria is also possible. However, PrP^Sc^ is only directly observable within the cytoplasm after disruption of proteostasis systems (Homma et al., 2014). Although it is still possible that the lower respiration rates observed in mitochondria isolated from prion-infected animals is the result of PrP^Sc^ interacting with mitochondria, it is also possible that accumulation and/or degradation of PrP^Sc^ during infection drives changes in protein expression that indirectly alter mitochondrial function (Ward et al., 2022).

In this study, we directly investigated the ability of two different mouse-adapted prion strains, 22L and 87V, to disrupt mitochondrial function in neural cells that do or do not express PrP^C^. The 22L and 87V strains were previously found to differ in structural stability, cellular degradation rates, pathology and disease incubation times (Shoup and Priola, 2021, 2023; Bett et al., 2012), which made them good candidates for possessing different capacities for disrupting cellular fitness. Our data show that during initial exposure to cells, only 22L PrP^Sc^ was able to alter mitochondrial redox potential and activity. We found that inhibiting endolysosomal acidification, which precedes PrP^Sc^ disassembly by the cell (Caughey et al., 1991; Shoup and Priola, 2021), disrupted the ability of 22L to alter mitochondrial redox state, suggesting that lysosomal degradation of PrP^Sc^ is important. Interestingly, in cells chronically infected with 22L prions, mitochondrial respiration was not significantly impacted despite signs of increased mitochondrial sensitivity to stress, suggesting that chronically infected cells are either more resistant to any damage triggered by PrP^Sc^ accumulation or might adapt to support persistent prion infection. Taken together, these data show that PrP^Sc^ triggers mitochondrial dysfunction in a prion strain-dependent manner that is facilitated by lysosomal processing, suggesting that cellular attempts to clear infectious prions might contribute to prion-mediated changes in mitochondria.

## RESULTS

### Mitochondrial redox state is altered during internalization of 22L but not 87V prions

Cellular internalization of non-prion amyloid fibrils can lead to changes in mitochondrial function, with potentially dire consequences for cellular fitness (Nagakannan et al., 2021; Umeda et al., 2011). As aberrant mitochondrial activity has also been observed in prion-infected animals (Faris et al., 2017b), we wanted to ascertain whether cellular internalization of PrP^Sc^ from two different prion strains, 22L and 87V, correlated with changes in mitochondrial function. We initially tested whether internalization of 22L and 87V PrP^Sc^ altered mitochondrial redox potential using a mitochondrial-localizing redox-sensitive green fluorescent protein (Mito-RoGFP) (Fig. S1). Changes in the redox state around Mito-RoGFP can be monitored by taking the ratio of excitation peaks at 406 and 469 nm (Fig. 1A). This ratio increases when the probe is exposed to reducing environments and decreases with exposure to oxidizing environments such as those induced by dithiothreitol (DTT) and hydrogen peroxide (H_2_O_2_), respectively (Fig. 1B). To better discriminate between mitochondrial dysfunction produced by internalized PrP^Sc^ and newly formed PrP^Sc^, we performed assays in a mouse neural cell line (CF10) that can internalize PrP^Sc^ (Greil et al., 2008; Shoup and Priola, 2021) but does not make PrP^C^ (Fig. 1C) (Greil et al., 2008), as well as in CF10 cells that produce mouse PrP^C^ engineered to express the anti-hamster PrP^C^ 3F4 antibody epitope (CF10-Mo3F4) (McNally et al., 2009; Choi and Priola, 2013). The PrP^C^ produced by CF10-Mo3F4 cells is converted into PrP^Sc^ by both 22L and 87V, and CF10-Mo3F4 cells can be infected by 22L but not 87V prions (McNally et al., 2009; Choi and Priola, 2013). The redox state of mitochondria was tracked over 24 h in Mito-RoGFP CF10 (Fig. 1C) and Mito-RoGFP CF10-Mo3F4 (Fig. 1D) cells after exposure to 0.25% normal brain homogenate (NBH), 22L brain homogenate (BH) or 87V BH. Compared to exposure to NBH, exposure to 22L BH led to development of an oxidizing environment in cellular mitochondria that peaked at 8 h regardless of whether the cells expressed PrP^C^, whereas exposure to 87V BH had no impact on mitochondrial redox state in either cell type (Fig. 1C,D).

**Fig. 1.**
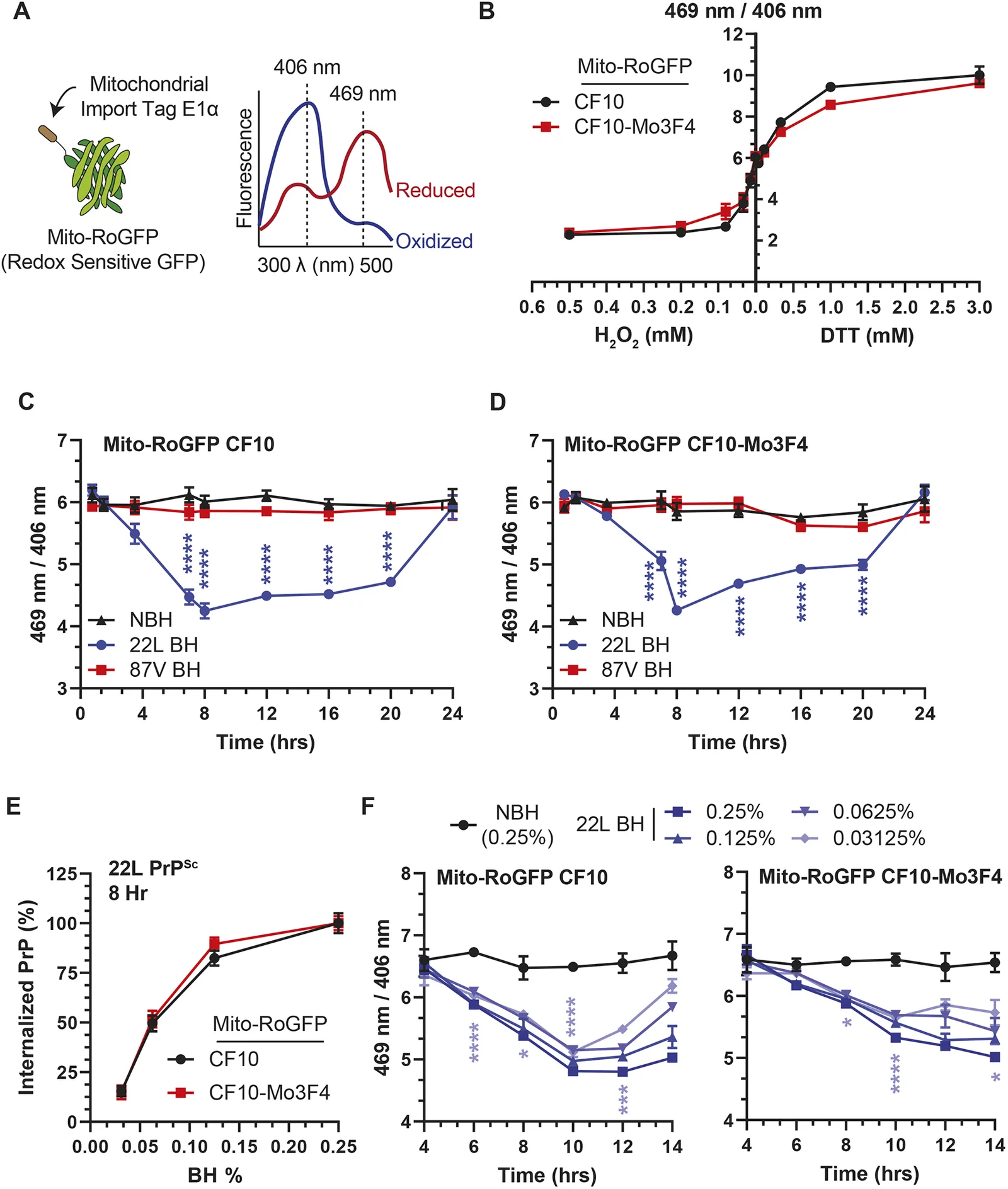
**Mitochondria take on a more oxidizing environment after exposure to 22L PrP^Sc^ BH.** (A) A redox state-sensitive mitochondrially localizing fluorescent protein known as Mito-RoGFP was used to monitor the redox state of mitochondria during PrP^Sc^ exposure. Left: graphic of Mito-RoGFP with a mitochondrial import tag. Right: excitation profile of Mito-RoGFP and its responsivity to changes in redox state. The ratio of fluorescence from excitation at 406 and 469 nm can be used to track the changes in redox state around the fluorescent protein. (B) Mito-RoGFP-expressing CF10 (black lines and circles) and CF10-Mo3F4 (red lines and squares) cells were treated with either 0.01–0.5 mM H_2_O_2_ or 0.01-3.0 mM dithiothreitol (DTT). Line plots show the average (±s.e.m.) 469/406 nm fluorescence ratio for *n*=24 replicates. (C,D) Mito-RoGFP-expressing (C) CF10 or (D) CF10-Mo3F4 cells were incubated with 0.25% normal brain homogenate (NBH) from C57BL/10 mice (black lines and triangles), and either 22L (blue lines and circles) or 87V (red lines and squares) brain homogenate (BH) for 24 h. Line plots show the average (±s.e.m.) of the 469/406 nm ratio for *n*=16 replicates for both cell types. (E) The amount of 22L PrP^Sc^ taken up by CF10 (black lines and circles) and CF10-Mo3F4 (red lines and squares) cells was determined by western blotting after exposure to 0.03–0.25% 22L BH for 8 h. The line plot shows the average (±s.e.m.) percentage of PrP^Sc^ taken up by the cell as pixel sum values normalized to the amount of PrP^Sc^ internalized after exposure to 0.25% BH for *n*=3 replicates. (F) Mito-RoGFP expressing CF10 (left) and CF10-Mo3F4 (right) cells were exposed to 0.03–0.25% 22L BH (shades of blue) and Mito-RoGFP fluorescence was monitored from 4 to 14 h after BH exposure. The line plots show the average (±s.e.m.) 469/406 nm ratio for *n*=8 replicates. For panels C–F, statistical significance was calculated by two-way ANOVA using Tukey's multiple comparisons test. The blue asterisks represent the statistical significance of cellular samples exposed to 22L BH compared to cells exposed to NBH. *P=0.01–0.05; ***P=0.001; ****P≤0.0009.

As 22L-infected brains contain approximately four times the amount of PrP^Sc^ as that in 87V-infected brains (Fig. S2), we wanted to determine how PrP^Sc^ levels influenced the ability of 22L BH to alter mitochondrial redox state. We first evaluated the amount of 22L PrP^Sc^ internalized by Mito-RoGFP CF10 cells, which produce redox-sensitive GFP but not PrP^C^, or Mito-RoGFP CF10-Mo3F4 cells, which produce redox-sensitive GFP and mouse PrP^C^ containing the 3F4 epitope (Mo3F4), after exposure to different concentrations of 22L BH for 8 h. As expected, western blot analysis of cell lysates from both cell lines showed that increasing amounts of 22L BH led to increasing levels of internalized PrP^Sc^ (Fig. 1E). We then exposed the cells to decreasing concentrations of 22L BH and used Mito-RoGFP to track changes in mitochondrial redox state from 4 to 14 h (Fig. 1F). In cells exposed to 22L, significant changes in mitochondrial redox state were seen at concentrations of 22L BH (0.0625%), in which the amount of PrP^Sc^ was the same or lower than in tests with 87V BH (compare Fig. 1F to Fig. 1C,D). By 14 h, the mitochondrial redox state had returned to near-normal levels at the lowest BH concentrations used, but only in cells that did not express PrP^C^ (Fig. 1F, compare left and right panels). Overall, the data suggest that the lack of an effect with 87V was not solely the result of a lower PrP^Sc^ concentration.

We next wanted to verify whether the change in mitochondrial redox potential was the direct result of 22L PrP^Sc^ as opposed to another molecule found in the 22L BH. To do this, we immunodepleted 22L PrP^Sc^ from 22L-infected BH using the 6H10 antibody (Fig. S3), which directly binds to PrP^Sc^ but not PrP^C^ (Horiuchi et al., 2009). Mito-RoGFP CF10 cells and Mito-RoGFP CF10-Mo3F4 cells were then exposed to either supernatants from the 6H10-immunodepleted 22L BH or control supernatants from antibody-free immunodepleted 22L BH and NBH (Fig. 2). After monitoring Mito-RoGFP fluorescence from 6 to 12 h, control immunodepleted supernatants of 22L BH led to significant oxidation of mitochondria, whereas no change in redox potential was observed in samples in which 6H10 was used to immunodeplete 22L PrP^Sc^ from infected BH. These data strongly suggest that 22L PrP^Sc^ was driving the changes in mitochondrial redox state observed during prion exposure.

**Fig. 2.**
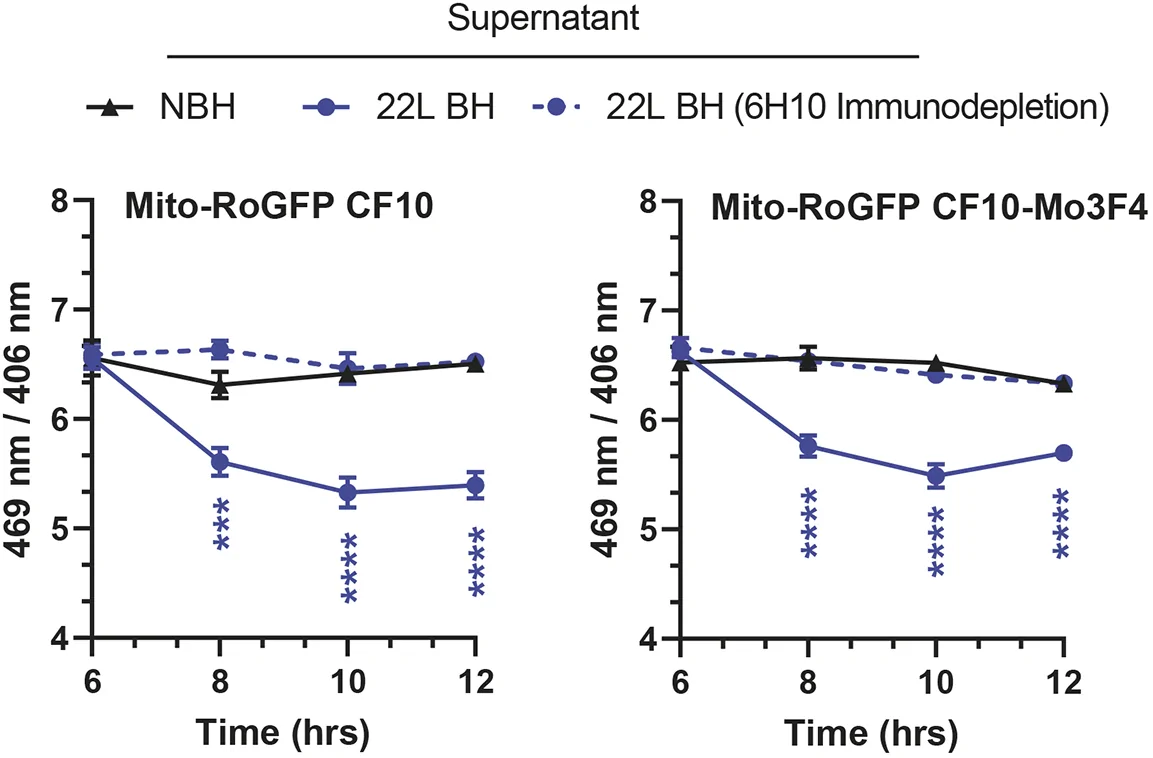
**Change in mitochondrial redox state is caused by 22L PrP^Sc^.** 22L BH was depleted of PrP^Sc^ using the 6H10 antibody. Mito-RoGFP CF10 (left) and CF10-Mo3F4 (right) cells were then exposed to either the BH supernatant devoid of 22L PrP^Sc^ (dashed blue line and circles), the no-antibody C57BL/10 NBH immunodepleted supernatant (solid black lines and triangles), or the no-antibody 22L BH immunodepleted sample (solid blue lines and circles). The fluorescence excitation ratio for Mito-RoGFP was determined at 6–12 h post exposure. All plots show the average (±s.e.m.) 469/406 nm ratio for *n*=8 replicates. Statistical significance was calculated by two-way ANOVA using Tukey's multiple comparisons test with the blue asterisks representing significance of the no-antibody 22L BH sample compared to NBH alone. ***P=0.002; ****P≤0.0009.

### Sarkosyl treatment interferes with the ability of 22L PrP^Sc^ to alter mitochondrial redox state

Previous work has found that treatment with sarkosyl, a detergent that can strip membrane from PrP^Sc^ (Benilova et al., 2020), reduces the neurotoxicity of PrP^Sc^ (Benilova et al., 2020), and we considered that sarkosyl treatment might also disrupt the ability of PrP^Sc^ to alter mitochondrial redox state. To test this, we exposed Mito-RoGFP CF10 and Mito-RoGFP CF10-Mo3F4 cells to sarkosyl-treated or untreated 0.25% NBH, 22L BH and 87V BH (Fig. 3A) and monitored the mitochondrial redox state 4–12 h post exposure. Although sarkosyl treatment did not completely inhibit the ability of 22L PrP^Sc^ to alter mitochondrial redox state, it did significantly reduce 22L-mediated redox changes in the mitochondria of both cell types (Fig. 3A). The shift in the ability of 22L to alter mitochondrial redox state after sarkosyl treatment was not the result of an inability of cells to take up 22L PrP^Sc^ from sarkosyl-treated BH (Fig. S4). These data suggest that membrane removal via sarkosyl treatment disrupts some property of PrP^Sc^ that drives redox state changes in mitochondria.

**Fig. 3.**
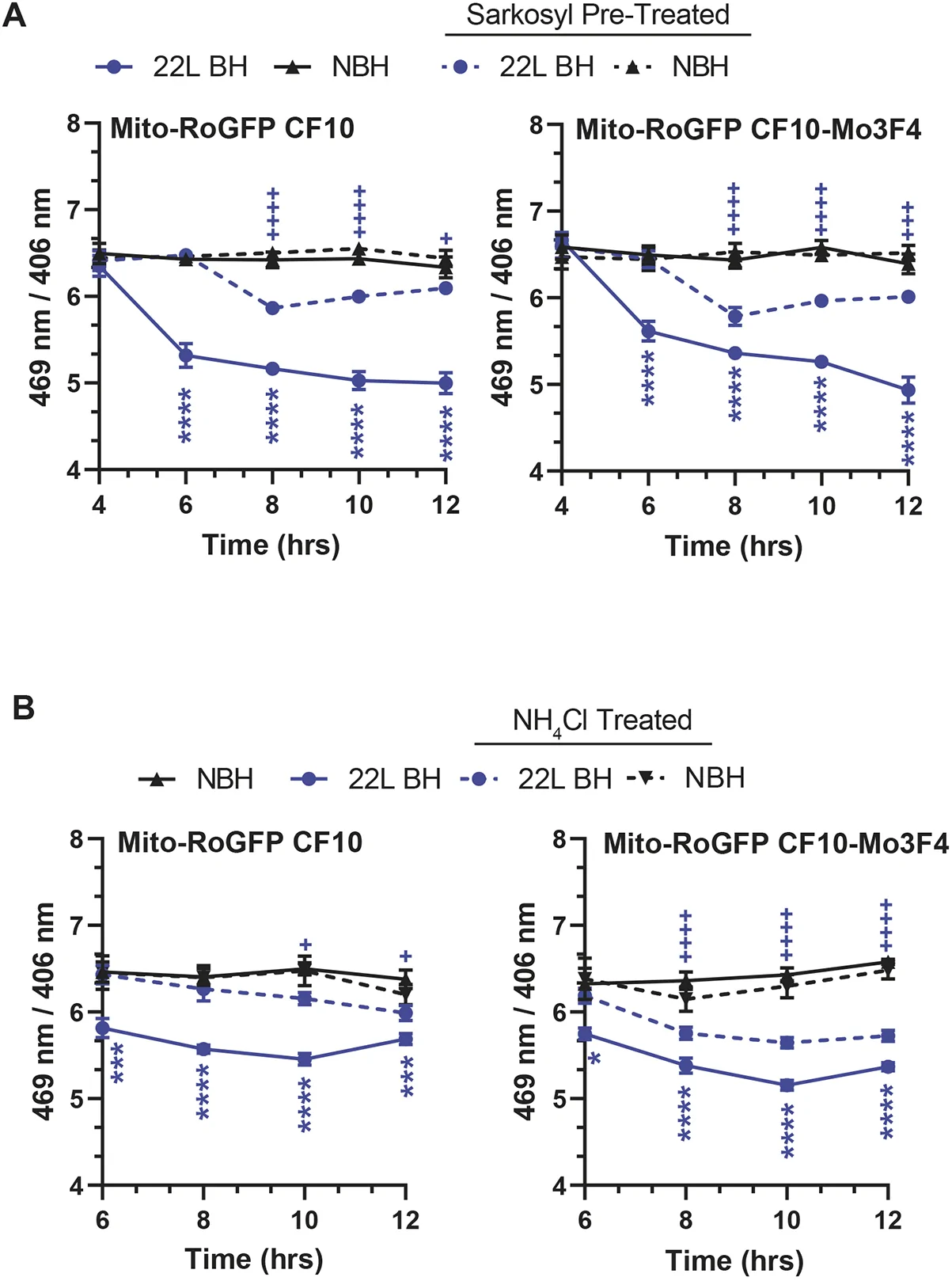
**Treatment with either sarkosyl or ammonium chloride reduces the ability of 22L PrP^Sc^ to alter mitochondrial redox state.** (A) Mito-RoGFP CF10 (left) and CF10-Mo3F4 (right) cells were treated with either C57BL/10 NBH (black lines and triangles) or 22L BH (blue lines and circles) that had (dashed lines) or had not (solid lines) been treated with sarkosyl. Cells were exposed to 0.25% BH for 4–12 h. (B) Mito-RoGFP CF10 (left) and CF10-Mo3F4 (right) cells were treated with either 0.25% NBH (black lines and triangles) or 22L BH (blue lines and circles) in the presence (dashed lines) or absence (solid lines) of ammonium chloride (NH_4_Cl). Cells were analyzed at 6–12 h post exposure. The line plots show the average (±s.e.m.) 469/406 nm ratio for *n*=8 replicates. Statistical significance was calculated by two-way ANOVA using Tukey's multiple comparisons test. Designated *P*-values are for NBH versus 22L BH samples and are indicated by blue asterisks for untreated samples and blue plus signs for sarkosyl- or NH_4_Cl-treated samples. *P=0.03; ***P=0.001–0.004; ****P≤0.0009; **^+^***P*=0.01–0.05; **^+++^***P*=0.003; **^++++^***P*≤0.0009.

### Endolysosomal acidification plays a role in 22L PrP^Sc^-mediated changes in mitochondrial redox state

As the structure of PrP^Sc^ can be disrupted by low pH (Shoup and Priola, 2024) and many lysosomal enzymes require a lower pH environment to function properly (Caughey et al., 1991), we next considered that lysosomal acidification might play a role in the mitotoxic properties of 22L PrP^Sc^. To test whether lysosomal acidification plays a role in the ability of 22L PrP^Sc^ to alter mitochondrial redox state, we exposed cells that did and did not make PrP^C^ to 0.25% NBH or 22L BH with and without ammonium chloride (NH_4_Cl) (Fig. 3B), which buffers endolysosomal vesicles, inhibiting endolysosomal acidification (Fig. S5). Although the effect was more significant in cells expressing PrP (Fig. 3B, right panel), treatment of cells with NH_4_Cl significantly reduced the ability of 22L PrP^Sc^ to oxidize mitochondria in both cell types, with no effect in cells exposed to NBH (Fig. 3B). Thus, the data indicate that endolysosomal acidification facilitates the mitotoxic properties of 22L PrP^Sc^.

### Exposure to prions alters mitochondrial respiration in a strain-dependent manner

In order to better understand how prions might influence mitochondrial activity, we analyzed changes in mitochondrial function using the Seahorse XFe96 Analyzer to monitor mitochondrial respiration (Fig. 4A) in Mito-RoGFP CF10 (Fig. 4B) and Mito-RoGFP CF10-Mo3F4 (Fig. 4C) cells exposed to 0.25% NBH, 22L BH or 87V BH for 24 h. Changes in mitochondrial function after exposure to 22L BH and 87V BH were compared to those following exposure to NBHs from the C57BL/10 mouse line (used to derive 22L BH) and from the VMDK mouse line (used to derive the 87V BH). NBHs from both strains of mice acted similarly and had no impact on mitochondrial function (Fig. S6); thus, for most experiments, only results for NBH derived from C57BL/10 mice are shown.

**Fig. 4.**
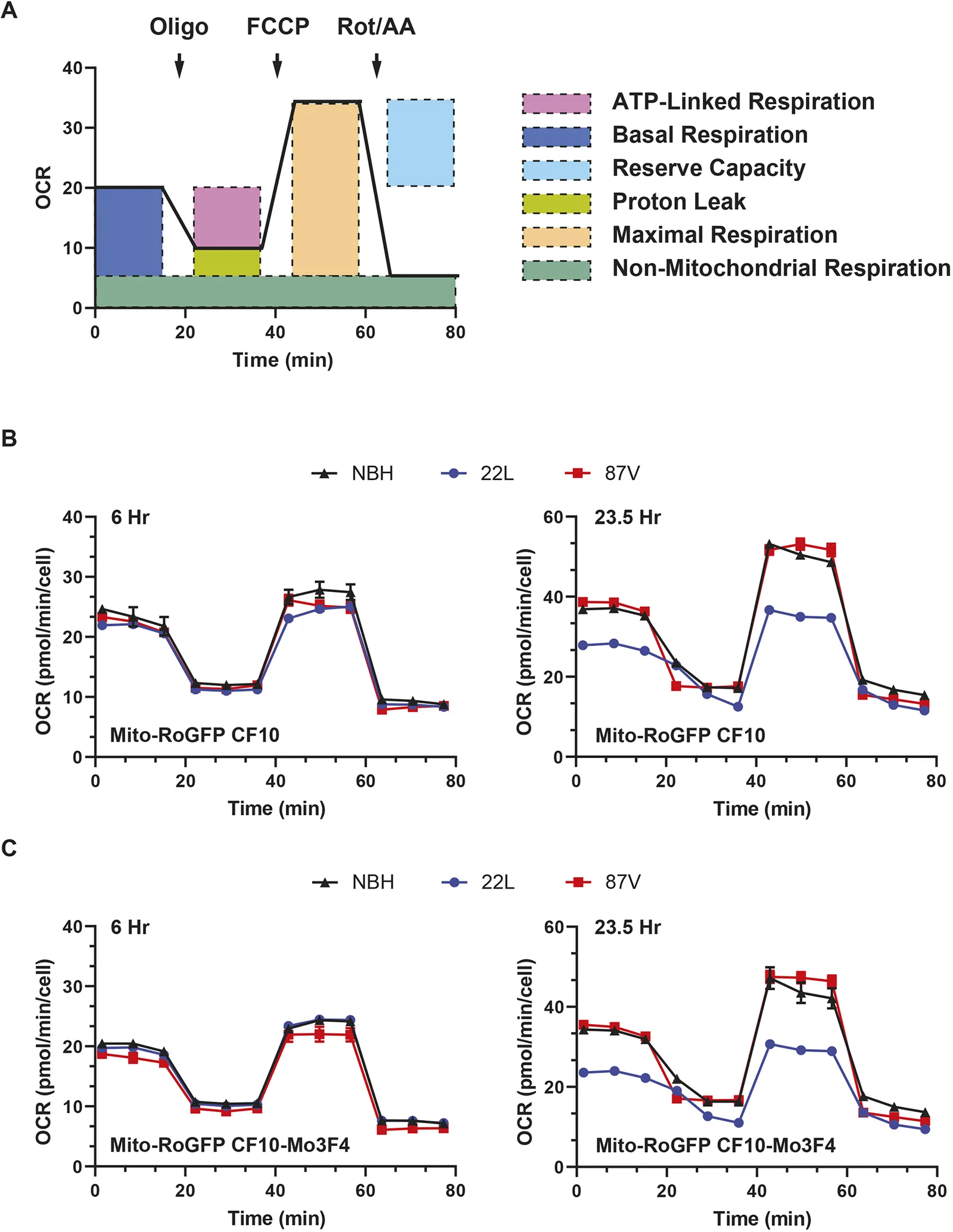
**Exposure to 22L prions leads to an overall reduction in mitochondrial respiration.** (A) Graphical representation of mitochondrial respiration assay data indicating how respiration (black line) changes as a result of exposure to oligomycin (oligo), carbonyl cyanide *p*-trifluoromethoxyphenylhydrazone (FCCP), and rotenone/antimycin A (Rot/AA), as well as how these data can be used to calculate different states of mitochondrial respiration as indicated by the different colored blocks (legend on the right). OCR, oxygen consumption rate. (B,C) Mito-RoGFP CF10 (B) and CF10-Mo3F4 (C) cells were exposed to 0.25% C57BL/10 NBH (black lines and triangles), 22L BH (blue lines and circles) or 87V BH (red lines and squares) for 6 h (left) or 23.5 h (right) and the OCR in picomoles (pmol) per minute (min) was measured using the Seahorse XFe96 Analyzer. The OCR was normalized to cell number and all plots show the average (±s.e.m.) OCR in pmol/min/cell for *n*=8 replicates.

Fig. 4 shows representative 6 and 23.5 h timepoints from the assay, whereas individual Seahorse assays for the remaining timepoints are shown in Fig. S7 (Mito-RoGFP CF10 cells) and Fig. S8 (Mito-RoGFP CF10-Mo3F4 cells). For both cell lines, by the 6 h timepoint, exposure to neither 22L BH nor 87V BH had induced a change in mitochondrial respiration compared to exposure to NBH (Fig. 4B,C, left panels). However, between 8.5 and 16 h, there appeared to be a significant drop in mitochondrial respiration in cells exposed to 22L BH but not to 87V BH (Figs S7 and S8), which continued through the final 23.5 h timepoint (Fig. 4B,C, right panels). These trends generally held true for both cell types and all timepoints assayed after 8 h, with mitochondrial respiration trending lower in cells exposed to 22L prions and remaining largely unchanged in cells exposed to 87V prions (Figs S7 and S8). As both Mito-RoGFP CF10 and Mito-RoGFP CF10 Mo3F4 cells produced similar responses to uptake of 22L, the impact of 22L on mitochondria was not the result of the process used to reintroduce Mo3F4 PrP^C^ into CF10 cells. In addition, expression of vector alone had no impact on mitochondrial respiration in the CF10 cell line (Fig. S9).

These data suggested that the impact of prion exposure on mitochondrial function increased over time and was dependent upon prion strain. We therefore determined how basal respiration, proton leak, reserve capacity, ATP-linked respiration, maximal respiration and non-mitochondrial respiration changed over time in response to prion exposure for each timepoint as shown in Fig. 4, Fig. S7 (Mito-RoGFP CF10) and Fig. S8 (Mito-RoGFP CF10-Mo3F4). Basal respiration reflects mitochondrial respiration to meet the normal energy demand of the cell, and reserve capacity indicates the spare oxidative capacity of the cell in response to increased energy demands or stress. ATP-linked respiration measures the oxygen consumed for ATP production, and proton leak indicates oxygen consumption not tied to ATP production. Maximal respiration is the highest possible rate of oxygen consumption, and non-mitochondrial respiration measures oxygen consumption from sources independent of the mitochondria.

Consistent with the data shown in Fig. 4, Figs S7 and S8, prior to 8 h post exposure to prion-infected BH, both cell types showed similar trends in mitochondrial respiration following exposure to 22L BH (Figs 5 and 6). Between 8 and 12 h post exposure, both cell types showed a consistent and significant decrease in all aspects of mitochondrial activity compared to that in cells exposed to NBH (Figs 5 and 6, blue lines). This was followed over the next 12 h by a gradual increase in oxygen consumption in Mito-RoGFP CF10 cells exposed to 22L (Fig. 5). This gradual increase was not observed in Mito-RoGFP CF10-Mo3F4 cells, in which oxygen consumption plateaued around 17 h and then began to decrease again (Fig. 6), suggesting that expression of PrP^C^ had a negative impact on the ability of mitochondria to recover from prion exposure.

**Fig. 5.**
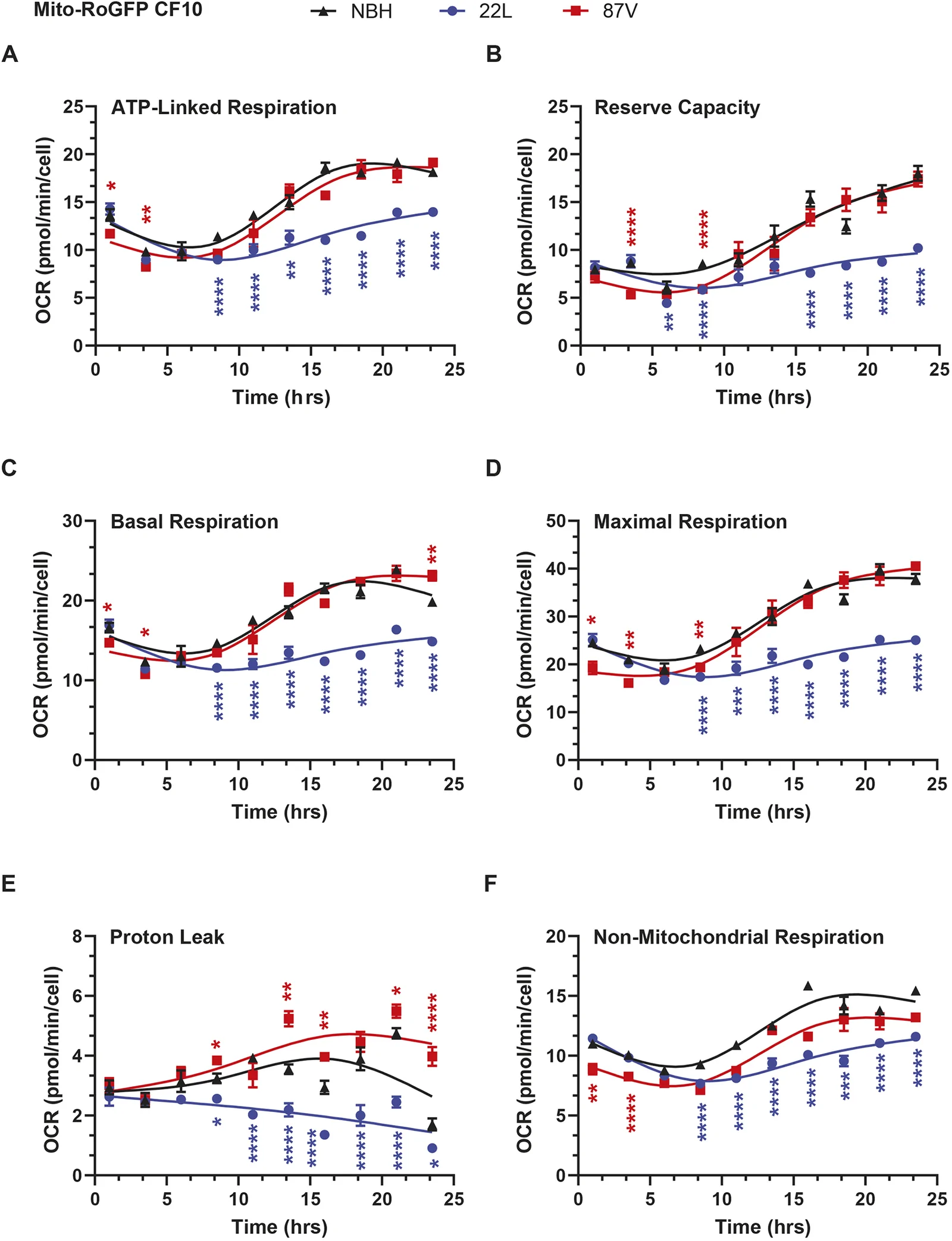
**Exposure to 22L prions reduces mitochondrial respiration in CF10 cells.** Mitochondrial oxygen consumption rate (OCR) of Mito-RoGFP CF10 cells treated with oligomycin, FCCP and rotenone/antimycin A (Fig. 4 and Fig. S7) was used to calculate mitochondrial respiration states: (A) ATP-linked respiration, (B) reserve capacity, (C) basal respiration, (D) maximal respiration, (E) proton leak and (F) non-mitochondrial respiration. Cells were exposed to 0.25% C57BL/10 NBH (black lines and triangles), 22L BH (blue lines and circles) or 87V BH (red lines and squares) for 24 h and mitochondrial OCR in picomoles (pmol) per minute (min) was assayed approximately every 2.5 h. The OCR was normalized to cell number and all plots show the average (±s.e.m.) OCR in pmol/min/cell for *n*=8 replicates. Lines are fits to data points that were calculated and drawn in GraphPad Prism as described in the Materials and Methods. Statistical significance was calculated by two-way ANOVA using Tukey's multiple comparisons test with the prion sample BH compared to the NBH sample indicated by asterisk color. *P=0.01–0.04; **P=0.05–0.09; ***P=0.001–0.004; ****P≤0.0009.

**Fig. 6.**
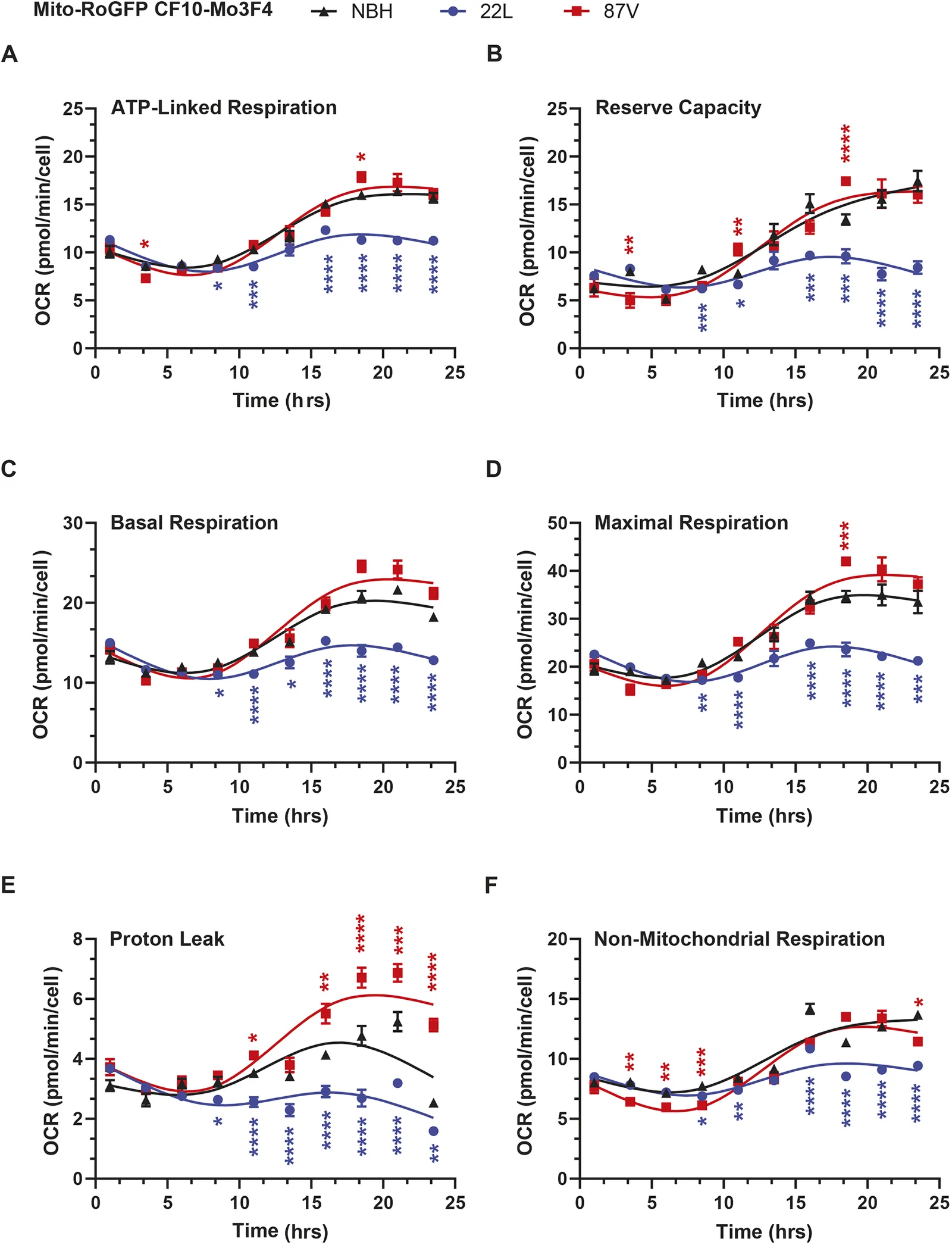
**PrP^C^ expression does not alter strain-dependent impacts on mitochondrial respiration.** Mitochondrial oxygen consumption rate (OCR) of Mito-RoGFP CF10-Mo3F4 cells treated with oligomycin, FCCP and rotenone/antimycin A (Fig. 4 and Fig. S8) was used to calculate mitochondrial respiration states: (A) ATP-linked respiration, (B) reserve capacity, (C) basal respiration, (D) maximal respiration, (E) proton leak and (F) non-mitochondrial respiration. Cells were exposed to 0.25% C57BL/10 NBH (black lines and triangles), 22L BH (blue lines and circles) or 87V BH (red lines and squares) for 24 h and mitochondrial OCR in picomoles (pmol) per minute (min) was assayed approximately every 2.5 h. The OCR was normalized to cell number and all plots show the average (±s.e.m.) OCR in pmol/min/cell for *n*=8 replicates. Lines are fits to data points that were calculated and drawn in GraphPad Prism as described in the Materials and Methods. Statistical significance was calculated by two-way ANOVA using Tukey's multiple comparisons test with the prion sample BH compared to the NBH sample indicated by asterisk color. *P=0.01–0.04; **P=0.05–0.09; ***P=0.001–0.004; ****P≤0.0009.

By contrast, mitochondrial respiration in both cell types following exposure to 87V BH was generally indistinguishable from that of cells exposed to NBH (Figs 5 and 6). Exposure to 87V BH induced a slight but significant difference in mitochondrial respiration compared to that following exposure to NBH between 0 and 5 h (Figs 5A–D and 6A,B). After 8 h, cells exposed to 87V were largely indistinguishable from cells exposed to NBH, with significant increases in maximal respiration and reserve capacity only observed in Mito-RoGFP CF10-Mo3F4 cells between 18 and 22 h. Interestingly, however, exposure of cells to 87V BH led to an increase in proton leak, whereas exposure to 22L BH led to a reduction in proton leak (Figs 5E and 6E), suggesting that there are prion strain-specific effects on mitochondrial oxygen consumption that occur in the absence of ATP production.

As the overall concentration of PrP^Sc^ in 87V-infected brains is roughly 25% of the amount in 22L-infected brains (Fig. S2), we wanted to see whether raising the concentration of 87V BH fourfold would result in a change in mitochondrial activity. We again exposed Mito-RoGFP CF10 and CF10-Mo3F4 cells to NBH and 87V BH, but at 0.25 and 1% concentrations. Despite the increase in PrP^Sc^ concentration, exposure to 87V still had little or no impact on mitochondrial respiration, with an increase in proton leak and only a small difference in non-mitochondrial respiration at 24 h (Fig. S10). Overall, our data indicate that the ability of prions to alter mitochondrial activity is strain specific, with 22L prions having a much more significant impact than 87V prions in depressing mitochondrial respiration, the extent of which correlates at least in part with lack of PrP^C^ expression.

Finally, in order to verify that changes in mitochondrial respiration were the direct result of 22L PrP^Sc^, the 6H10 antibody was used to immunodeplete PrP^Sc^ in 22L BH before adding the PrP^Sc^-depleted BH to Mito-RoGFP CF10 cells. After 21 h, cellular respiration was analyzed using the Seahorse XFe96 Analyzer (Fig. S11). Consistent with the results of the PrP^Sc^ immunodepletion in Fig. 2, removal of 22L PrP^Sc^ from BH disrupted the ability of 22L to alter mitochondrial respiration during prion exposure. BH cleared of detectable 22L PrP^Sc^ acted similarly to control NBH samples, whereas 22L BH treated with beads only (i.e. no 6H10 antibody) disrupted mitochondrial respiration to the same extent as untreated 22L BH (Fig. S11).

### Cells persistently infected with 22L prions are more sensitive to oxidative stress

Previous studies have found that gene expression profiles change during chronic prion infection (Moody et al., 2009; Sorce et al., 2020), possibly to help cells adapt to prion infection. Cells persistently infected with prions, which maintain infectious prions through continuous passage, are more sensitive to oxidative stress than uninfected cells (Haigh et al., 2011; Milhavet et al., 2000). In addition, *in vivo* studies have shown that mitochondrial respiration can be impaired during prion infection (Faris et al., 2017b; Choi et al., 2014). As exposure of cells to 22L prions drives changes in mitochondrial redox state (Figs 1–3) and respiration (Figs 4–6), we wanted to know whether mitochondrial integrity was still disrupted during persistent prion infection. We therefore tested whether mitochondrial respiration in CF10-Mo3F4 cells persistently infected with 22L prions was also impaired. These cells maintain 22L PrP^Sc^ over multiple passages following exposure to 22L BH through the continuous conversion of PrP^C^ (McNally et al., 2009) (Fig. 7A; Fig. S12). Interestingly, we found no difference in mitochondrial activity between CF10-Mo3F4 cells persistently infected with 22L prions and uninfected CF10-Mo3F4 cells (Fig. 7B).

**Fig. 7.**
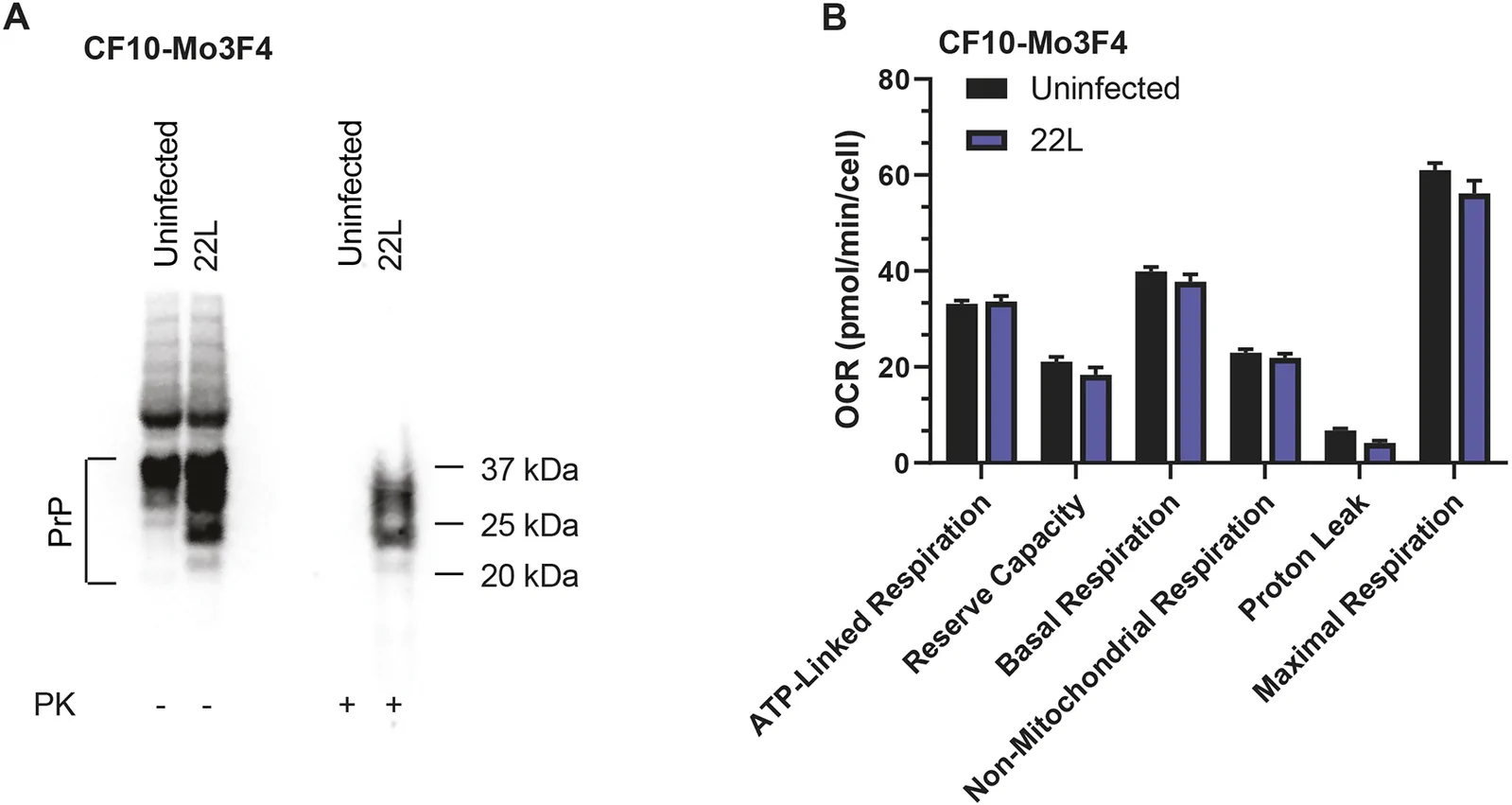
**Mitochondrial respiration is unaltered in cells persistently infected with 22L.** (A) Western blots of lysates from uninfected or 22L-infected CF10-Mo3F4 cells that were (+) or were not (−) treated with proteinase K (PK). The blot was developed with the anti-PrP 6D11 monoclonal antibody. Actin loading controls are shown in Fig. S12. Molecular mass markers are shown on the right. The western blot was performed once. (B) Mitochondrial respiration was analyzed in 22L-infected (blue bars) and uninfected (black bars) CF10-Mo3F4 cells using the Seahorse XFe96 Analyzer. Mitochondrial oxygen consumption rates (OCR) in picomoles (pmol) per minute (min) were normalized to the number of cells and used to calculate ATP-linked respiration, reserve capacity, basal respiration, non-mitochondrial respiration, proton leak, and maximal respiration as described in the Materials and Methods. The bar plot shows average (±s.e.m.) OCR in pmol/min/cell for *n*=24 replicates. Statistical significance was calculated by two-way ANOVA using Tukey's multiple comparisons test. No significant differences were observed in mitochondrial respiration between uninfected and 22L prion-infected CF10-Mo3F4 cells.

We next considered that mitochondrial function in 22L-infected CF10-Mo3F4 cells could be more sensitive to oxidative stress, as increased sensitivity to oxidative stress has been reported for 22L-infected hypothalamic neuronal GT1 cells (Milhavet et al., 2000). To test this, we exposed 22L-infected and uninfected CF10-Mo3F4 cells to 0.5 mM H_2_O_2_ for 60 min, conditions that can significantly impact mitochondrial function (Fig. S13), and then analyzed mitochondrial respiration using the Seahorse XFe96 Analyzer. The data showed a significant reduction in the mitochondrial activity of H_2_O_2_-treated 22L-infected CF10-Mo3F4 cells compared to that of uninfected cells (Fig. 8A), with significant reductions in basal, maximal and non-mitochondrial respiration (Fig. 8B). Interestingly, there was essentially no mitochondrial spare respiratory capacity in prion-infected cells exposed to H_2_O_2_ (Fig. 8B), suggesting that persistent 22L prion infection reduced the ability of the cell to respond to increases in cellular energy demands following stress. These data indicate that, although cells persistently infected with prions might recover from or eventually adapt to prion-associated mitochondrial dysfunction, they nonetheless suffer from a significantly reduced capacity for responding to oxidative stress.

**Fig. 8.**
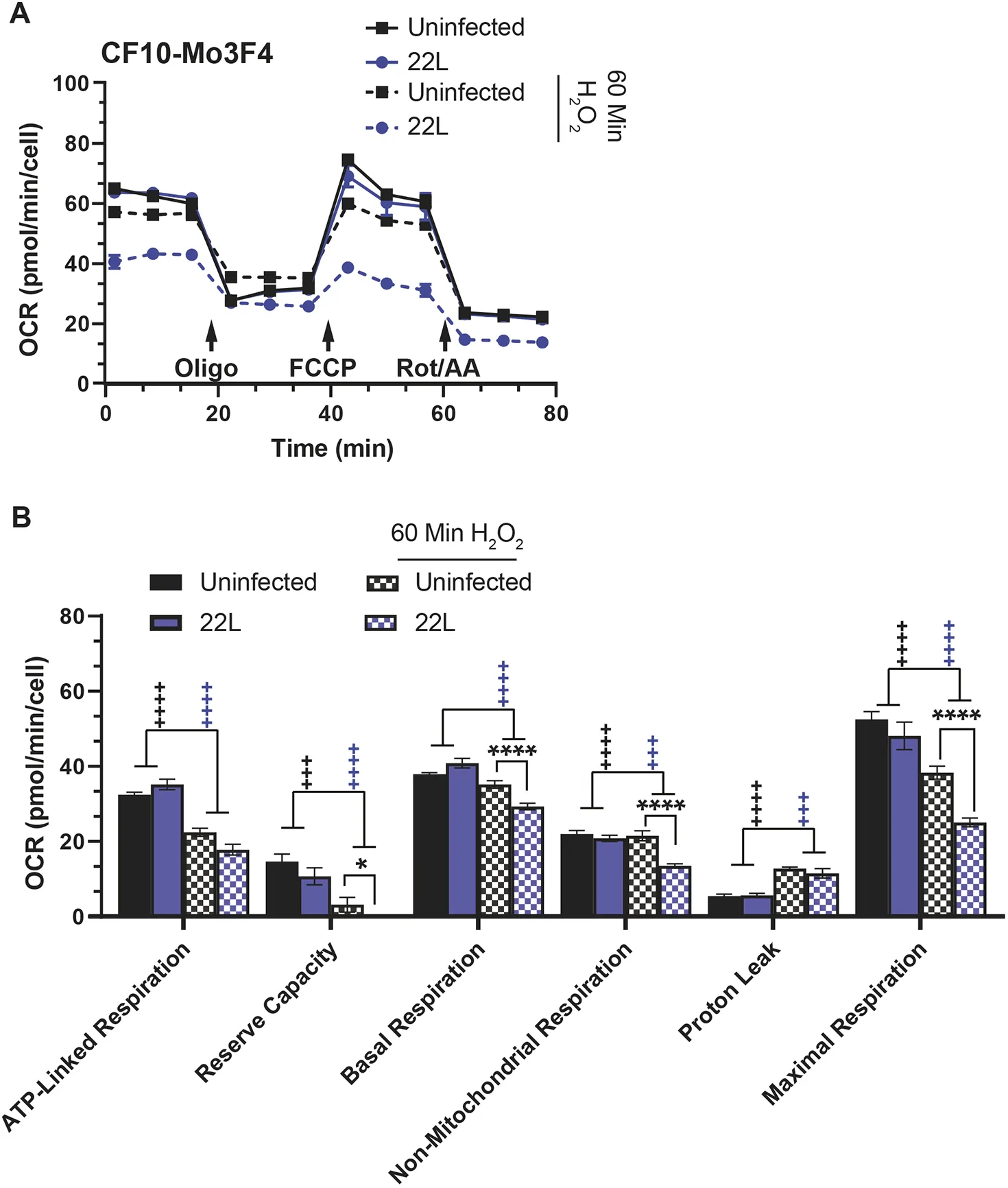
**Cells persistently infected with 22L prions have impaired mitochondrial respiration when stressed.** (A) Infected (blue lines and circles) and uninfected (black lines and squares) CF10-Mo3F4 cells were (dashed lines) and were not (solid lines) stressed by exposure to 0.5 mM hydrogen peroxide (H_2_O_2_) for 60 min. Mitochondrial activity was then analyzed over 80 min using the Seahorse XFe96 Analyzer. The oxygen consumption rate (OCR) in picomoles (pmol) per minute (min) normalized to cell number is shown over time. Oligo, oligomycin; Rot/AA, rotenone/antimycin A. (B) OCR data in panel A were used to calculate mitochondrial fitness as in Fig. 7. Solid black bars show uninfected cells, solid blue bars show 22L-infected cells, black checkered bars show H_2_O_2_-treated uninfected cells and blue checkered bars show H_2_O_2_-treated 22L-infected cells. Data shown represent the average (±s.e.m.) OCR in pmol/min/cell of *n*=24 replicates. Statistical significance was calculated by two-way ANOVA using Tukey's multiple comparisons test. In panel B, black asterisks indicate the difference between H_2_O_2_-treated uninfected and 22L-infected CF10-Mo3F4 cells, whereas plus symbols indicate the difference between H_2_O_2_-treated and untreated uninfected (black plus signs) or 22L-infected (blue plus signs) CF10-Mo3F4 cells. *P=0.01–0.04; ****P≤0.0009; **^+++^***P*=0.001–0.005; **^++++^***P*≤0.0009.

## DISCUSSION

The 22L prion strain is structurally less stable, degraded more slowly in the lysosome and produces a significantly faster disease progression in mice than the 87V prion strain (Shoup and Priola, 2021, 2023; Bett et al., 2012; Priola, 2018; Choi and Priola, 2013). It is not currently understood why disease duration varies between prion strains, but it might result from strain-dependent differences in strain propagation rate and/or the ability of the strains to interfere with critical cellular processes. In this study, we investigated the ability of 22L and 87V prions to alter both mitochondrial redox state and respiration in the neural cell line CF10 and found that only exposure to 22L PrP^Sc^ produced large changes in mitochondrial redox state and respiration. Removal of PrP^Sc^ from 22L-infected BH disrupted the ability of 22L prions to alter both mitochondrial redox state and respiration, strongly indicating that 22L PrP^Sc^ in the BH was the primary driver of the changes observed in the cell. The inability of 87V prions to induce changes in mitochondrial redox state indicates that not all prion strains are capable of inducing mitochondrial dysfunction and might help to explain the shorter disease incubation times in 22L-infected mice compared to those in 87V-infected mice.

In previous work, we found that degradation of 22L PrP^Sc^ taken up by CF10 cells produced partial truncation products roughly 23 kDa in size that were not seen after uptake of 87V PrP^Sc^, indicating that cellular processing of 22L PrP^Sc^ produces unique degradation products (Shoup and Priola, 2023). These partial truncation products were detected within the same timeframe (4 to 24 h) during which we detected changes in mitochondrial redox state and respiration, raising the intriguing possibility that truncated PrP molecules may trigger mitochondrial dysfunction if they are capable of exiting the lysosome. Indeed, when we disrupted endolysosomal acidification, a step previously found to be critical for the degradation of PrP^Sc^ (Shoup and Priola, 2021), we found that the ability of 22L PrP^Sc^ to alter mitochondrial redox potential was reduced (Fig. 3B). There is evidence that accumulation of β-2 microglobulin (B2M), A-β (amyloid β) and α-synuclein (SNCA) fibrils disrupt the lysosomal membrane and exit the lysosome, inducing mitochondrial dysfunction (Okoshi et al., 2015; Umeda et al., 2011; Grassi et al., 2018). In the case of α-synuclein, this is associated with a specific α-synuclein degradation product that is released from the lysosome that then travels to and interacts with the mitochondria (Grassi et al., 2018). Our data are consistent with 22L PrP^Sc^ acting similarly, and we hypothesize that lysosomal processing facilitates the ability of 22L to alter mitochondrial redox state by potentially releasing PrP^Sc^ degradation products lacking the GPI anchor that might directly or indirectly drive mitochondrial dysfunction.

In this study, we observed that sarkosyl treatment of 22L PrP^Sc^ also greatly reduced its ability to alter mitochondrial redox state (Fig. 3A), indicating that PrP^Sc-^mediated disruption of mitochondria requires membrane anchoring. Similar observations were found in other works, which showed that the removal of membrane from PrP^Sc^ with sarkosyl pretreatment disrupted the neurotoxicity of PrP^Sc^ (Benilova et al., 2020), indicating that membrane association is generally important for prion-mediated disruption to cellular fitness. Previous work found that sarkosyl treatment also disrupted formation of 23 kDa 22L PrP^Sc^ (Shoup and Priola, 2023), a finding consistent with our hypothesis that PrP^Sc^ degradation products are important in prion-mediated mitochondrial dysfunction. Although it is not well understood how association between PrP^Sc^ and membrane might impact mitochondrial fitness, it has been postulated that membrane-anchored PrP^Sc^ can twist membranes and alter membrane rigidity around PrP^Sc^ in a way that induces membrane tears (Kraus et al., 2021). PrP^Sc^ might also potentially permeabilize endolysosomal vesicles in a similar manner to how aggregates of tau (MAPT) and α-synuclein disrupt membranes in Alzheimer's disease and Parkinson's disease, respectively (Rose et al., 2024; Freeman et al., 2013). However, we also found that buffering lysosomal acidification reduces the ability of 22L to impact mitochondria, indicating that membrane anchoring and lysosomal activity might be part of a process that allows 22L to disrupt mitochondrial function. Tearing or permeabilization of lysosomal membranes by membrane-anchored PrP^Sc^ could thus provide a means by which PrP^Sc^ or its degradation products, with or without the GPI anchor, could be released into the cytoplasm to interact with mitochondria and impact their ability to function normally.

During exposure to 22L PrP^Sc^, the redox-sensitive probe RoGFP detected a more oxidizing environment in mitochondria (Fig. 1). This could be indicative of an increase in reactive oxygen species (ROS), which has been found to occur during prion infection (Haigh et al., 2011). RoGFP detects changes in local redox state via cysteine bond formation on its surface, which shifts its fluorescence profile (Wagener et al., 2016). Reducing environments lead to less disulfide bond formation, whereas oxidizing environments lead to more disulfide bond formation. Although exposure to H_2_O_2_ does result in a drop in the ratio of absorbance at 469/406 nm, a sign of high disulfide bond formation on RoGFP and the presence of an oxidizing environment (Fig. 1B), the same shift can result from mitochondria losing their reducing capacity. NADH is a potent reducing agent in the mitochondria, which is converted into NAD^+^ during ATP production. A decrease in the ratio of NADH/NAD^+^ would also lower the reducing capacity of mitochondria and cause more disulfide bond formation in RoGFP. It is thus possible that a shift in specific mitochondrial metabolites such as NADH/NAD^+^ could elicit a similar impact on RoGFP as ROS.

We found that mitochondrial respiration in CF10 cells persistently infected with 22L prions was similar to that of uninfected cells (Fig. 7) and did not show the same impairment in mitochondrial function that was observed following initial exposure to 22L (Figs 4–6). This suggests that the cells might have adapted to prion infection. Strikingly, however, the persistently infected cells clearly had no reserve respiratory capacity in response to oxidative stress (Fig. 8). Cells infected with 22L have a much higher rate of mitophagy than uninfected cells (Kim et al., 2022), suggesting that they might be adapting to mitochondrial dysfunction during prion infection by increasing the rate at which damaged mitochondria are recycled. A similar tactic for dealing with mitochondrial dysfunction can be seen in other neurodegenerative diseases, such as Alzheimer's disease, in which induction of mitophagy improves average mitochondrial function in the cell (Sharma et al., 2022). Increased rates of mitophagy in prion-infected cells might help them maintain a healthy pool of mitochondria, leading to our observation of similar mitochondrial respiration with and without prion infection. However, increased levels of mitophagy might also push homeostasis and recycling systems to a point where they are more easily overwhelmed by any additional stress, which might be why prion-infected cells were so much more sensitive to H_2_O_2_-induced oxidative stress than uninfected cells.

## MATERIALS AND METHODS

### Animal care and prion propagation

Propagation of 22L and 87V prions was carried out previously (Shoup and Priola, 2023) in C57BL/10 and VMDK mice, respectively. Mice were euthanized after development of late-stage prion disease and the brains of infected and uninfected mice were harvested and stored at −80°C until use. The Rocky Mountain Laboratories Animal Care and Use Committee reviewed, approved and authorized all animal protocols, which covered inoculation, care into prion disease, disease endpoints and euthanasia (protocol number 020-2021). Animal care followed the Guide for the Care and Use of Laboratory Animals of the National Institutes of Health.

### BH preparation

As described previously (Shoup and Priola, 2021), uninfected as well as 22L- and 87V-infected mouse brains were thawed on ice and homogenized in phosphate-buffered saline (PBS) to a concentration of 10% (wt/vol) in a Mini-BeadBeater-8 instrument (Biospec). BHs were centrifuged at 500 ***g*** for 5 min to clarify samples before supernatants were removed, aliquoted, snap frozen in liquid nitrogen and stored at −80°C until use. Uninfected C57BL/10 and VMDK NBHs were used as negative controls for 22L and 87V prion-infected mice, respectively, and gave identical results in all experiments.

### Cell line formation, cell growth and plating

CF10 cells, a mouse neural cell line that does not express PrP^C^, were derived from transgenic mice in which expression of PrP^C^ was disrupted by insertion of a neomycin cassette into the open reading frame of the PrP gene *Prnp* (Greil et al., 2008; McNally et al., 2009). CF10-Mo3F4 cells (McNally et al., 2009) were derived by transducing CF10 cells with a non-replicating murine retrovirus carrying the gene for mouse PrP^C^ mutated to express the unique hamster PrP^C^ 3F4 antibody epitope (Mo3F4). These were the primary cell lines used in this work and were modified to constitutively express Mito-RoGFP proteins. The plasmid for Mito-RoGFP, pEGFP-N1 (BD Biosciences) (Hanson et al., 2004), was purchased from Addgene (plasmid number 82370). CF10 cells were transfected using Lipofectamine 3000 (Thermo Fisher Scientifc) and were sorted by fluorescence on a FACS flow cytometer (BD Biosciences) to isolate cells that permanently expressed Mito-RoGFP. Similarly to previous work, either mouse Mo3F4 PrP^C^ or the empty retroviral vector pSFF (Bestwick et al., 1988) was introduced into the genomes of CF10 cells expressing MitoRoGFP by transfection with non-replicating mouse retroviruses (Priola et al., 2001) to yield the cell lines Mito-RoGFP CF10-Mo3F4 or Mito-RoGFP CF10-pSFF, respectively. A FACS flow cytometer (BD Biosciences) was again used to sort cells so that prion protein-producing cell lines could be isolated. All cells were cultured in OptiMEM (Gibco) supplemented with 10% fetal bovine serum (FBS, ATLAS Biologicals), 100 μg streptomycin (Gibco) and 100 U of penicillin (Gibco), referred to as supplemented OptiMEM. Cells were grown to 95% confluency, following which they were resuspended in saline-trypsin-versine solution, diluted 1/10 to 1/5 in new medium and transferred to a new plate or flask. Cells were grown by incubation at 37°C with 5% ambient carbon dioxide in a humidified incubator.

Prior to every assay, cells were counted using an Accuri C6 flow cytometer (BD Biosciences) and plated so as to be either ∼80% confluent for RoGFP assays or ∼30% confluent for mitochondrial Seahorse assays. Cell concentration was determined by analysis of 50 μl of cell suspension. For Seahorse assays, cells were plated at 3×10^3^ cells/well in specialized Seahorse 96-well culture plates (Agilent). Cells used for RoGFP assays were plated into 96-well optical bottom plates (Nunc) at 2.6×10^4^ cells/well. After each Seahorse assay, relative differences in cell concentration between wells were determined by Hoechst 33342 (Thermo Fisher Scientific) fluorescence on a ClarioStar plate reader (BMG Labtech) with excitation and emission at 355 and 455 nm, respectively. Hoechst 33342 was added to Seahorse medium to a concentration of 1 μg/ml before being incubated with cells for 30 min at 37°C; the cells were then washed three times in Seahorse DMEM (Agilent) before fluorescence was measured. The fluorescence from each well was normalized to the mean and inserted into the Seahorse Wave software (Agilent) to correct for variation in cell concentration between wells.

### MitoTracker staining

Cells were plated on glass-bottomed 35 mm culture dishes (Mattek). To label cellular mitochondria, MitoTracker (Thermo Fisher Scientific) was diluted to 100 nM in supplemented OptiMEM and incubated with cells for 45 min at 37°C. Cells were then washed in OptiMEM to remove excess MitoTracker and images were acquired on a Zeiss LSM 880 microscope driven by ZEN v.2.3 software (Carl Zeiss Microscopy). Confocal *z*-stacks were acquired on the LSM 880 microscope using a Plan Apochromat 63×1.4 oil immersion lens with an oil immersion refractive index of 1.518 at a lateral resolution of 44 nm and *z*-spacing of 260 nm.

### Sarkosyl treatment

Prior to use, frozen BHs were thawed in a room-temperature water bath and then mixed by vigorous pipetting. Untreated BHs were diluted to the indicated concentration into supplemented OptiMEM. Media with and without BH were then placed over cells. Sarkosyl treatment of BH was carried out by first diluting 10% BH 1:1 with 4% sarkosyl (Milipore Sigma) in 1× PBS for a final concentration of 2% sarkosyl and 5% BH. Detergent treatment was carried out at 37°C for 30 min, following which the BH was centrifuged at 100,000 ***g*** in a 100.2 TLA rotor (Beckman) for 1 h to pellet PrP^Sc^. Supernatants were removed and pellets were washed in 1× PBS before being resuspended in a volume of 1× PBS that was half the starting volume, creating a solution of PrP^Sc^ with a concentration equivalent to 10% BH. Treated and untreated resuspended BH samples were then diluted into supplemented medium to the indicated concentration.

### PrP^Sc^ immunodepletion

Immunodepletions were performed by adding the 6H10 mouse monoclonal anti-PrP^Sc^ antibody (Horiuchi et al., 2009) into 10% BH to a final concentration of 0.1 mg/ml. BH was incubated with 6H10 for 2 h at 4°C on a rocker; then, 25 μl of protein G resin beads (Thermo Fisher Scientific) was added and the mixture was incubated for 1 h at 4°C with rocking. Beads were gently spun out of solution at 5000 ***g*** on a tabletop centrifuge (Thermo Fisher Scientific) alongside controls that received protein G beads but did not receive 6H10 antibody. This was repeated on the BH supernatant from each successive immunodepletion until PrP^Sc^ was no longer detected by western blotting (an additional three immunodepletion cycles); then, the PrP^Sc^-depleted BH supernatant was diluted into supplemented medium and added to cells.

### Mitochondria redox state assay with Mito-RoGFP

CF10 and CF10-Mo3F4 cells that expressed the Mito-RoGFP fluorescent protein were used to determine how prion BH influences mitochondrial redox state. Mito-RoGFP-expressing cells were exposed to sarkosyl-treated and untreated BH with and without 5 mM ammonium chloride for different lengths of time at 37°C. Cell medium was then removed and cells were washed three times with imaging buffer (50 mM HEPES pH 7.4, 130 mM sodium chloride, 5 mM potassium chloride, 10 mM D-glucose, 1 mM magnesium chloride, 1 mM calcium chloride). Then, 200 μl of imaging buffer was placed over cells and the 96-well plate was placed into the ClarioStar plate reader. Cells were excited at 469 and 406 nm and emission was recorded at 510–520 nm. Non-fluorescent CF10 and CF10-Mo3F4 cells that did not express Mito-RoGFP were used as controls for background fluorescence and the average fluorescence from these samples was subtracted from fluorescence signals emitted from cells expressing Mito-RoGFP. After the background fluorescence was subtracted from each measurement, the ratio of background-corrected fluorescence from each excitation wavelength was determined for each well. Ratios of emission after excitation at 469 and 406 nm were then averaged and s.e.m. values calculated.

### Seahorse-based mitochondrial respiration assay

CF10 and CF10-Mo3F4 cells, with or without Mito-RoGFP expression, were exposed to prion-infected or uninfected BHs and analyzed for changes in mitochondrial respiration using a Seahorse XFe96 Mito-Tox Assay kit (Agilent). CF10-Mo3F4 cells persistently infected with 22L prions were also analyzed. For cells exposed to BH at 37°C, BH was removed at different timepoints post exposure and cells were washed three times in Seahorse DMEM (Agilent) supplemented with 2 mM glutamine, 2 mM pyruvate and 10 mM D-glucose (Agilent). Cells were then covered in 180 μl of supplemented Seahorse DMEM before being analyzed in a Seahorse XFe96 analyzer. Before each run, plates were equilibrated for 30 min according to the manufacturer's instructions. Each experiment used a minimum of eight replicate wells per sample. Parameters for the assay were set to a 30 s mixing phase followed by a 10 s rest period and a 2 min read period for each measurement. This was repeated twice for basal readings and then twice after each injection. Cells were subjected to 1.75 μM oligomycin (Agilent), 2 μM carbonyl cyanide *p*-trifluoromethoxy phenylhydrazone (FCCP; Agilent) and 0.5 μM rotenone/antimycin A (Agilent), and the oxygen consumption rate (OCR) before and after exposure to each compound was determined. OCR was determined at three timepoints before treatment and at three timepoints after treatment for a total of 12 timepoints over ∼78 min. Basal respiration was determined using the last point before the addition of oligomycin. Respiration after oligomycin, FCCP and rotenone/antimycin A treatment was determined using the lowest, highest and lowest OCR values, respectively, in the set of three timepoints taken after each treatment.

To determine the concentration of H_2_O_2_ and exposure time that had the strongest effect on mitochondrial respiration, increasing concentrations of H_2_O_2_ were added to the medium of 22L-infected CF10-Mo3F4 cells. At different timepoints post exposure, the medium was removed, cells were washed with in Seahorse supplemented DMEM, and mitochondrial respiration was measured as described above. CF10-Mo3F4 cells persistently infected with 22L prions as well as uninfected CF10-Mo3F4 cells were then assayed following the same protocol and using the determined optimal concentration and exposure time of H_2_O_2_.

For all Seahorse assays, OCR was first corrected by subtracting the background (average OCR from wells without cells), and then non-mitochondrial respiration was subtracted from these values. These were the OCR numbers used for all subsequent calculations. Non-mitochondrial respiration was set as the lowest measured OCR after rotenone/antimycin A treatment and was subtracted from pre- and post-oligomycin treatment OCR values to determine basal and proton leak-related respiration, and from post-FCCP OCR values to determine maximal respiration. ATP-linked respiration was determined by subtracting the post-oligomycin treatment OCR from the pre-oligomycin treatment OCR, and reserve capacity respiration was determined by subtracting basal OCR from maximal respiration OCR. Values were independently calculated for each well and averaged with *n*-values indicated for each assay.

### Western blot analysis

Samples of cell lysates and prion-infected BHs were treated with 50 μg/ml of proteinase K (PK; EMD Millipore) for 1 h at 37°C before being mixed with 1 mM Pefabloc (Sigma-Aldrich) to inhibit PK digestion. Samples were then mixed with 4× NuPage sample buffer (Novex) to 1.5× before heat denaturation at 95°C for 5 min. Samples were run on NuPage 10% Bis-Tris gels (Novex) for 3 to 4 h at a constant 75 V. Gel contents were transferred to polyvinylidene difluoride (PVDF, EMD Milipore) Immobilon-P membranes with a Bio-Rad gel system run at a constant 34 V for 16 h. PVDF membranes were then blocked with a 5% powdered milk and 1× TBST solution [10 mM Tris HCl pH 8.0, 150 mM NaCl, 0.05% Tween 20, 5% powdered milk (wt/vol)] for 1 h at room temperature with gentle rocking. The anti-PrP antibody 6D11 (BioLegends, antibody epitope PrP amino acid residues 93–109) was diluted 1:10,000 in 1× TBST and incubated with the membranes for 1.5 h. Membranes were rinsed four times with 1× TBST over 30 min before being incubated with sheep anti-mouse secondary antibody conjugated to horseradish peroxidase (GE Amersham) at a 1:50,000 dilution for 1 h. Membranes were again rinsed four times with 1× TBST over 30 min before a final rinse in deionized H_2_O. Membranes were then treated with ECL Plus reagent (GE Healthcare Life Sciences) and imaged on an iBright imager (Thermo Fisher Scienific). The pixel intensity of prion protein bands was quantified using Un-Scan-IT software (version 7.1; Silk Scientific Corporation).

### Statistical analysis

Fluorescence ratios for RoGFP assays were calculated in Excel (Microsoft; version 2511) and median population fluorescence values were calculated in BD C Sampler Plus (BD Biosciences; version 1.0.34.1), with data normalization performed in Excel. Mitochondrial OCR values were also calculated in Excel but all data were ultimately imported into GraphPad Prism software (version 8.0.2) for data analysis. Prism was used to calculate s.e.m. values and perform the indicated tests for significance. In the figures, asterisks and plus signs represent *P*-values within specific ranges: *^/+^*P=*0.01–0.04; **^/++^*P*=0.005–0.009; ***^/+++^*P=*0.001–0.004; and ****^/++++^*P*≤0.0009.

## Supplementary Material

10.1242/joces.264762_sup1 Supplementary information
