# Supplementary material for: Exposure of cells to prions induces prion strain-dependent changes in mitochondrial redox state and respiration
Source: J Cell Sci. 2026 Sep 25;139(18):jcs264762. doi: 10.1242/jcs.264762 (PMC13680968; doi:10.1242/jcs.264762)
Supplement: Supplementary file 1 [file joces-139-264762-s1.pdf]

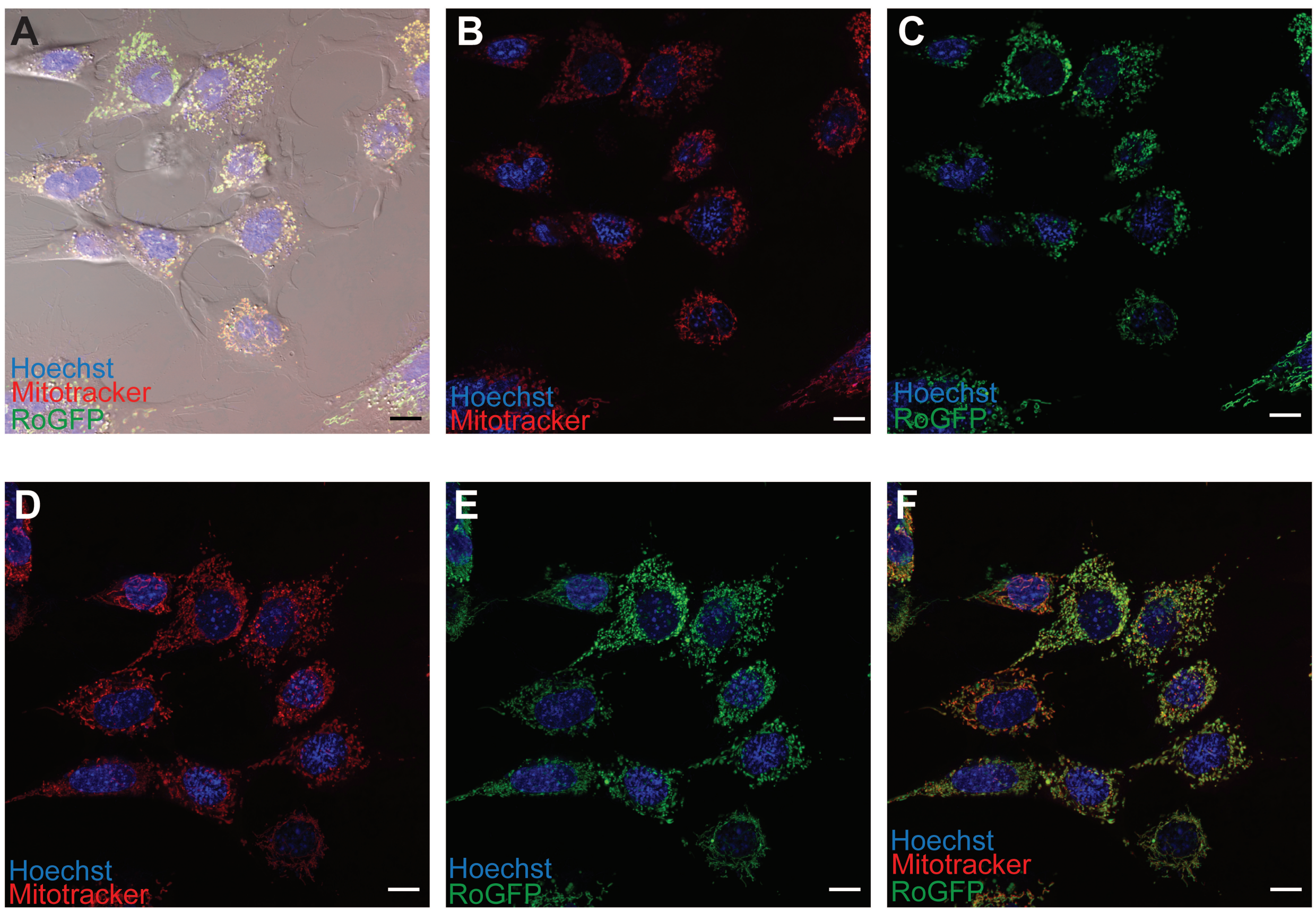

Fig. S1. Colocalization of Mitotracker and RoGFP in CF10 cells. (A) Overlay of differential interference contrast (DIC) and fluorescently labelled Mitotracker and RoGFP. (B) Red laser channel only (561nm) showing no bleed-through from the green laser channel (488nm). (C) Green laser channel only showing no bleed-through from the red laser channel. (D) Mitotracker (red) fluorescence. (E) RoGFP fluorescence. (F) Overlay image of panels D and E showing Mitotracker and RoGFP colocalization. Images represent all z-stack slices combined. For all panels, nuclei are stained with Hoechst stain (blue). Scale bar: 10 µm.

Journal of Cell Science • Supplementary information

A

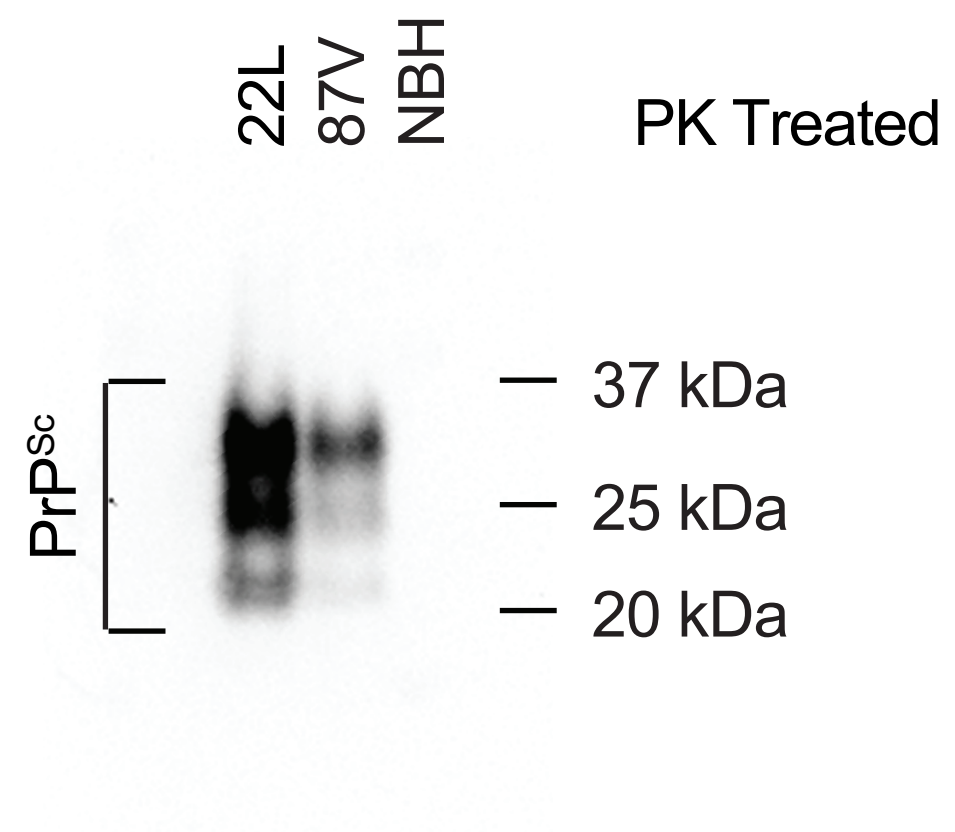

B

Strain	PrP^{Sc} / Actin Pixel Sum Ratio
22L	5.49
87V	1.39
Ratio of 22L to 87V PrP^{Sc}	3.95

C

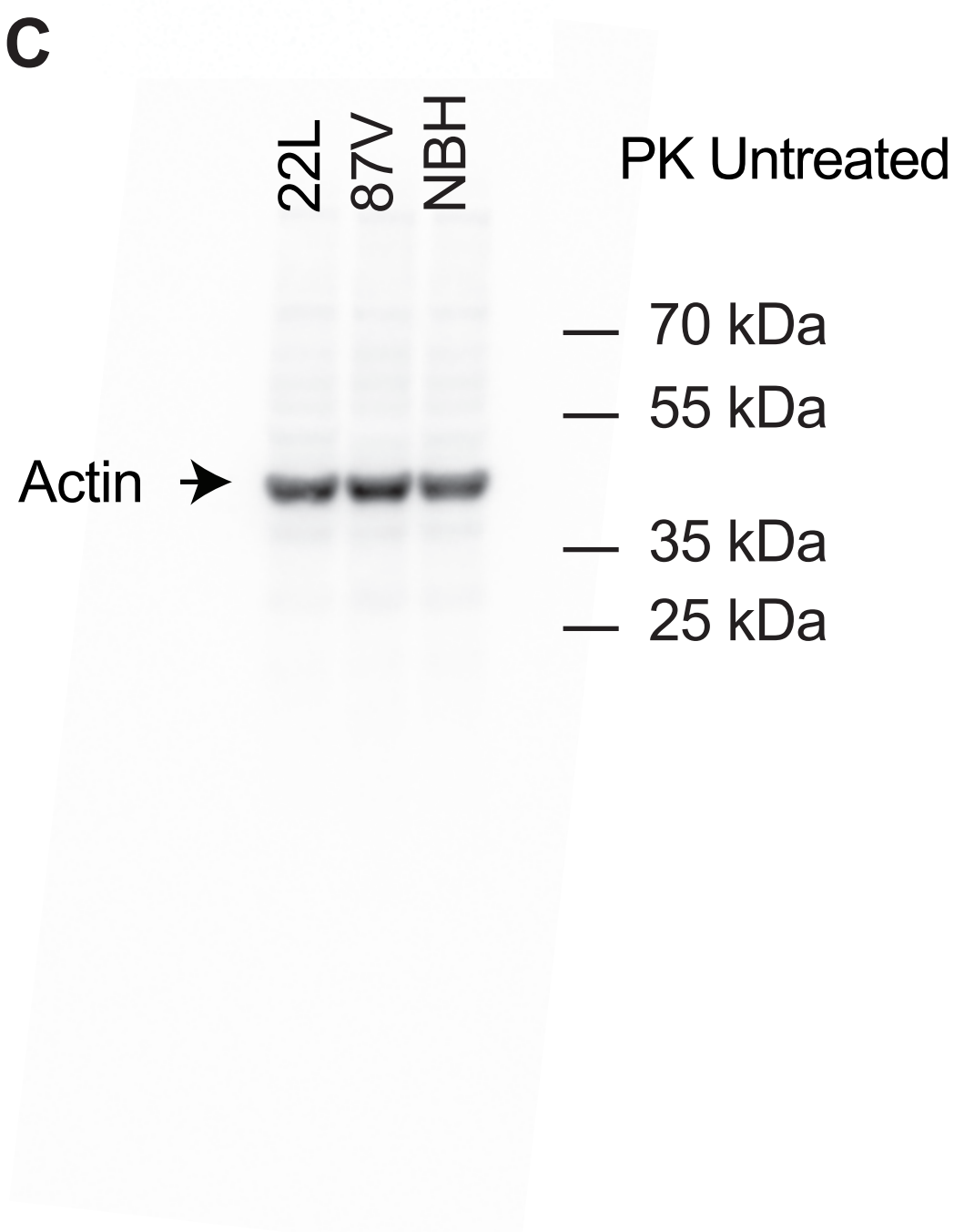

Fig. S2. Brain homogenates of 22L infected mice contain higher levels of PrP^{Sc} than brain homogenates from 87V infected mice. (A) PrP^{Sc} levels in 0.25% C57BL/10 normal brain homogenate (NBH), 22L brain homogenate (BH), and 87V BH. All samples were PK treated, denatured by boiling in SDS, and analyzed by SDS-PAGE and western blot using the anti-PrP 6D11 mouse monoclonal antibody which recognizes denatured 22L and 87 PrP^{Sc} equivalently. (B) Background corrected pixel intensity sums for PrP^{Sc} bands, normalized to background corrected pixel intensity sums for actin, in samples of 22L and 87V infected BH as well as the ratio of 22L and 87V pixel intensities. (C) Actin loading control for untreated brain homogenates from panel A. Molecular weight markers in kilodaltons (kDa) are indicated to the right of the blots.

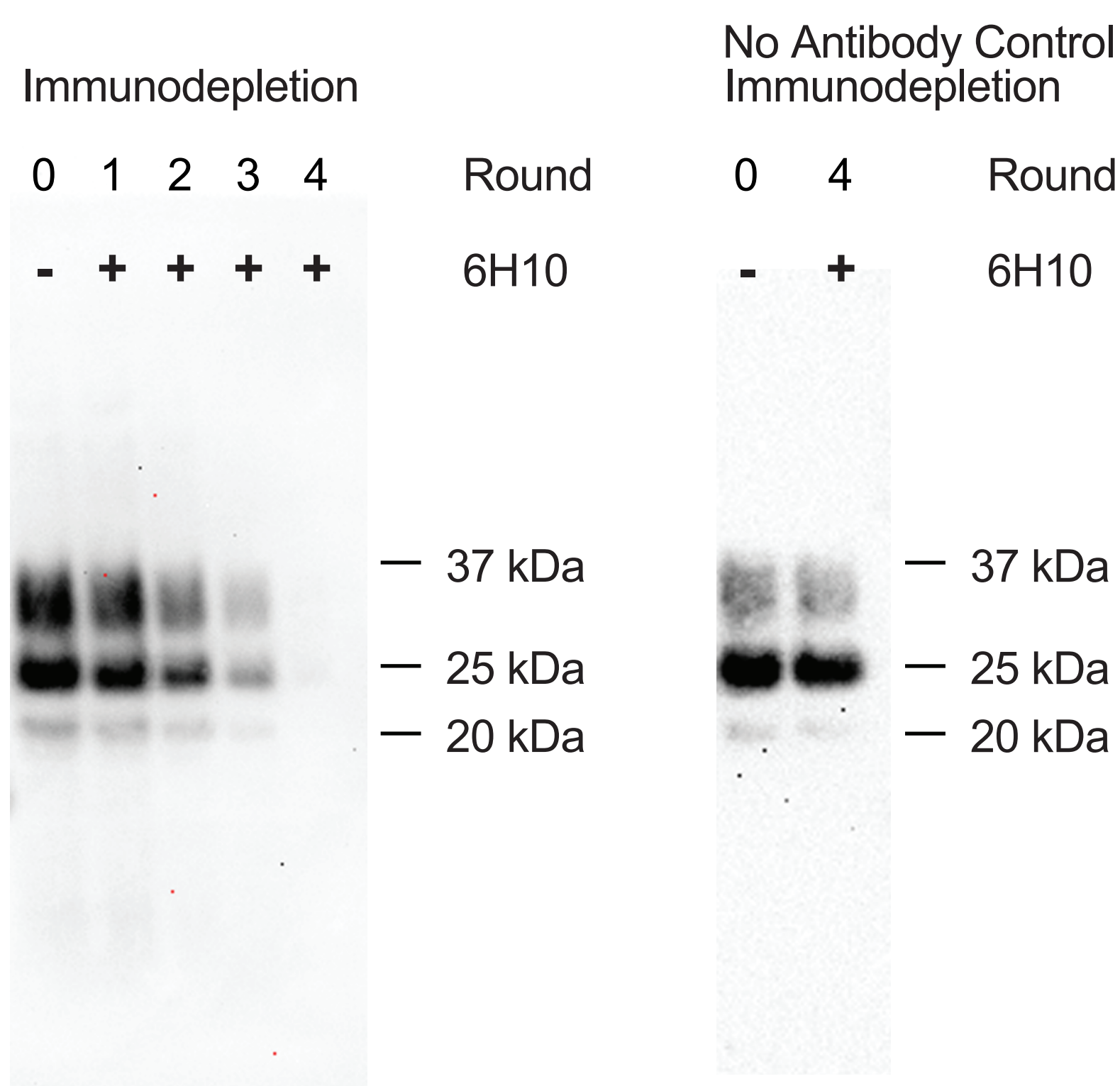

Fig. S3. Removal of PrP^{Sc} from 22L infected brain homogenate using the 6H10 antibody. Western blot of 22L PrP^{Sc} levels after incubation of BH with (+) and without (-) the 6H10 antibody. After each round (0-4) of incubation, 6H10 immunodepletion samples were PK treated and assayed for PrP^{Sc}. A parallel negative control immunodepletion was treated the same way (i.e. with four immunodepletions performed, 4th depletion shown), but with protein G beads only (no antibody was added). The western blot was developed using the anti-PrP 6D11 mouse monoclonal antibody. Molecular weight markers in kilodaltons (kDa) are indicated to the right of the blot shown.

Journal of Cell Science • Supplementary information

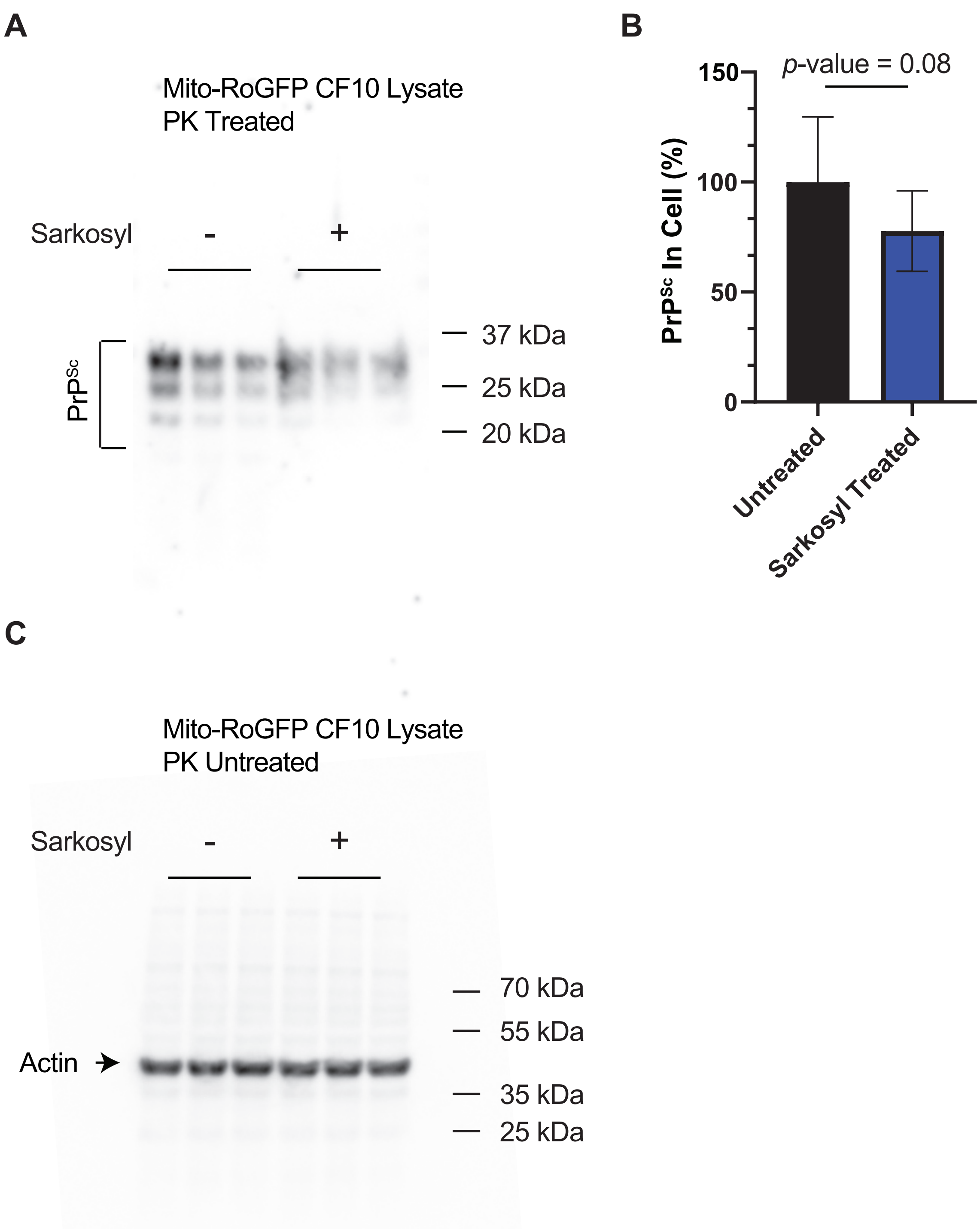

Fig. S4. Sarkosyl treatment does not significantly impact uptake of 22L PrP^{Sc}. (A) Western blots showing PrP^{Sc} levels in cells exposed to 0.25% 22L BH for 16 hrs that were (+) or were not (-) exposed to sarkosyl. The bracket indicates the three major PrP^{Sc} bands. (B) Percentage of PrP^{Sc} that was (blue bar) or was not (black bar) treated with sarkosyl and then taken up by the cell. The *p*-value was calculated using the paired Student's t-test in Graphpad Prism. Pixel sums of PrP^{Sc} were normalized to the Pixel sums of Actin. (C) Western blot showing actin (arrow) loading controls for data shown in panel (A). Molecular markers are indicated to the right of blots.

Journal of Cell Science • Supplementary information

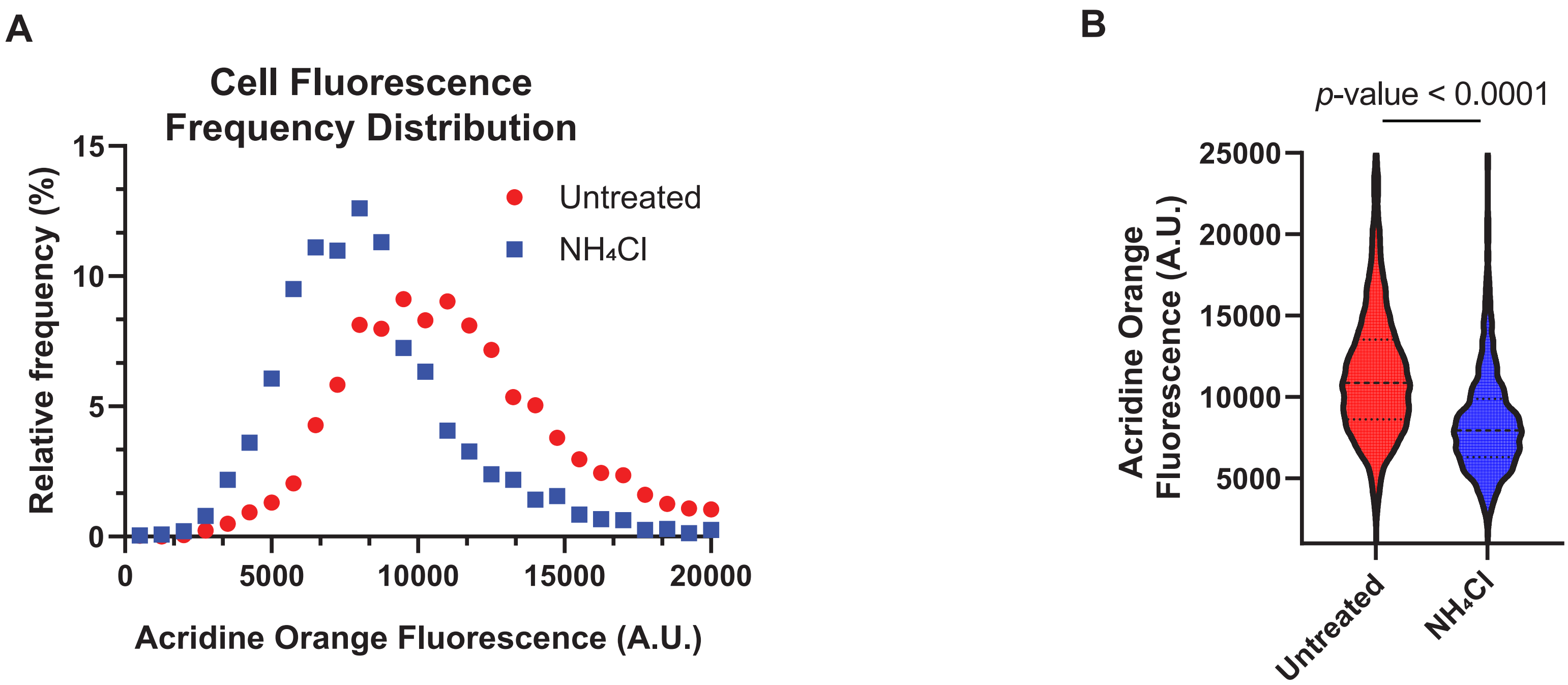

Fig. S5. Ammonium chloride reduces lysosomal pH after an hour of exposure. Characterization of lysosomal pH in Mito-RoGFP CF10 cells that had (blue squares or regions) or had not (red circles or regions) been exposed to ammonium chloride (NH_4Cl) for 1 hr. Cells were analyzed by flow cytometry with changes in the fluorescence of acridine orange indicating changes in lysosomal pH. (A) The frequency distributions and (B) violin plots shown are calculated from the fluorescence intensities of individual cells in three separate replicates (~10,000 cells/replicate). Calculations for frequency distributions and paired t-tests shown in the violin plots were calculated in Graphpad Prism. Arbitrary Units (A.U.)

Journal of Cell Science • Supplementary information

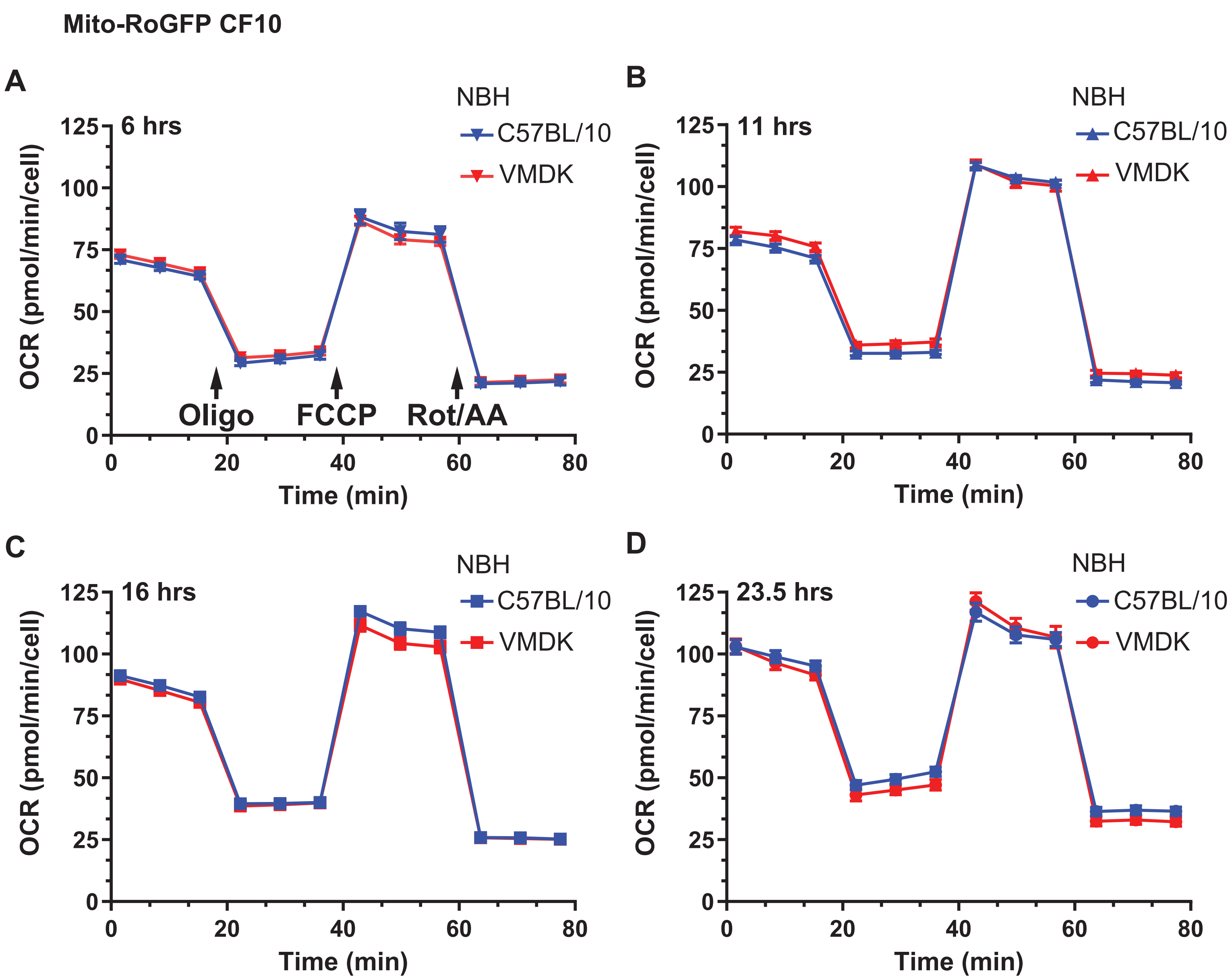

Fig. S6. Normal brain homogenates from C57BL/10 and VMDK mice have no impact on mitochondrial respiration. CF10 cells expressing Mito-RoGFP were exposed to 0.25% normal brain homogenate (NBH) from C57BL/10 (blue lines) and VMDK (red lines) mice. Oxygen consumption rate (OCR) was measured at (A) 6 (inverted triangles), (B) 11 (triangles), (C) 16 (squares), and (D) 23.5 (circles) hrs in picomoles (pmol) per minute (min) using the Seahorse XFe96 Analyzer. Cells were treated with oligomycin (Olig), FCCP, and rotenone/antimycin A (Rot/AA) to measure mitochondrial function. OCR was normalized to cell number. All plots show the average OCR in pmol/min/cell ± SEM for n=10 replicates.

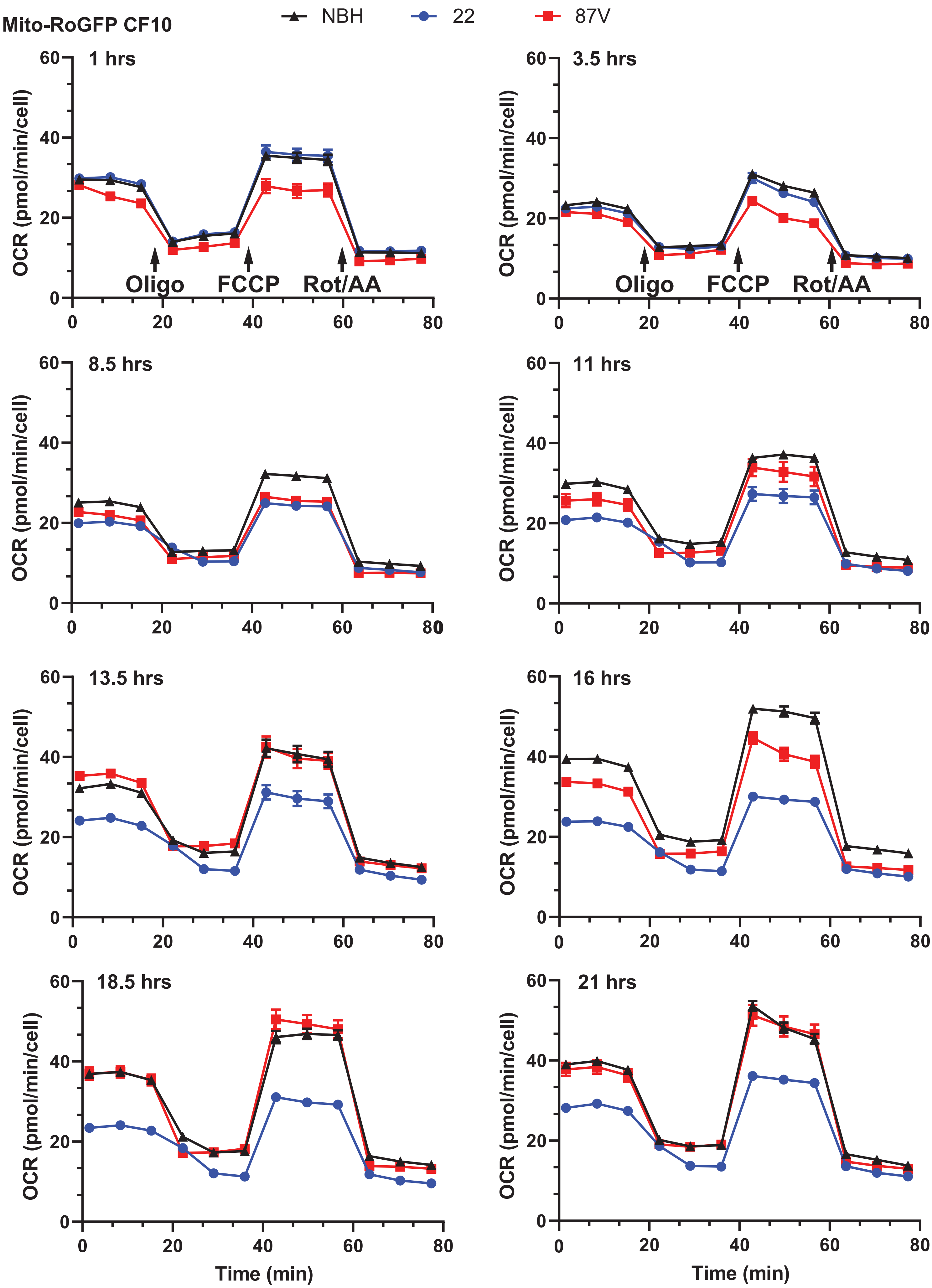
Mito-RoGFP CF10
NBH
22
87V
1 hrs
3.5 hrs
8.5 hrs
11 hrs
13.5 hrs
16 hrs
18.5 hrs
21 hrs
OCR (pmol/min/cell)
Oligo
FCCP
Rot/AA
Time (min)
0
20
40
60
80

Fig. S7. Exposure to 22L prions alters mitochondrial respiration over time. Mito-RoGFP expressing CF10 cells were exposed to 0.25% C57BL/10 Normal brain homogenate (NBH, black lines and triangles), 22L BH (blue lines and circles), or 87V BH (red lines and squares) for 1, 3.5, 8.5, 11, 13.5, 16, 18.5, and 21 hrs, and the oxygen consumption rate (OCR) in picomoles (pmol) per minute (min) was measured using the Seahorse XFe96 Analyzer. Cells were treated with oligomycin (oligo), FCCP, and rotenone/antimycin A (Rot/AA) as indicated. The OCR was normalized to cell number and all plots show the average OCR in pmol/min/cell ± SEM for n=8 replicates.

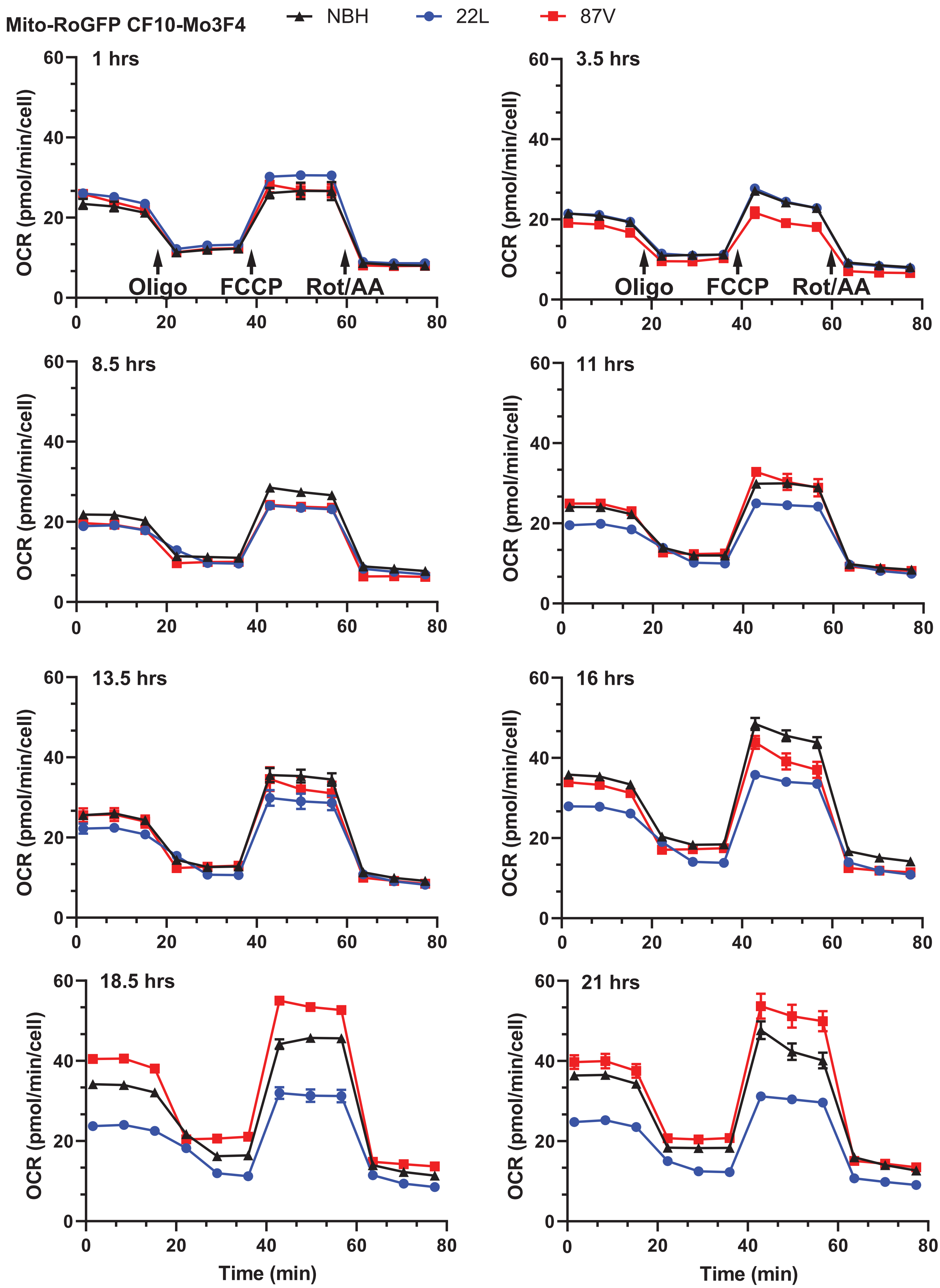

Journal of Cell Science • Supplementary information

Fig. S8. PrP^C expression does not alleviate 22L PrP^{Sc} mediated changes in mitochondrial respiration. Mito-RoGFP expressing CF10-Mo3F4 cells were exposed to 0.25% C57BL/10 Normal brain homogenate (NBH, black lines and triangles), 22L BH (blue lines and circles), or 87V BH (red lines and squares) for 1, 3.5, 8.5, 11, 13.5, 16, 18.5, and 21 hrs and the oxygen consumption rate (OCR) in picomoles (pmol) per minute (min) was measured using the Seahorse XFe96 Analyzer. Cells were treated with oligomycin (oligo), FCCP, and rotenone/ antimycin A (Rot/AA) as indicated. The OCR was normalized to cell number and all plots show the average OCR in pmol/min/cell + SEM for n=8 replicates.

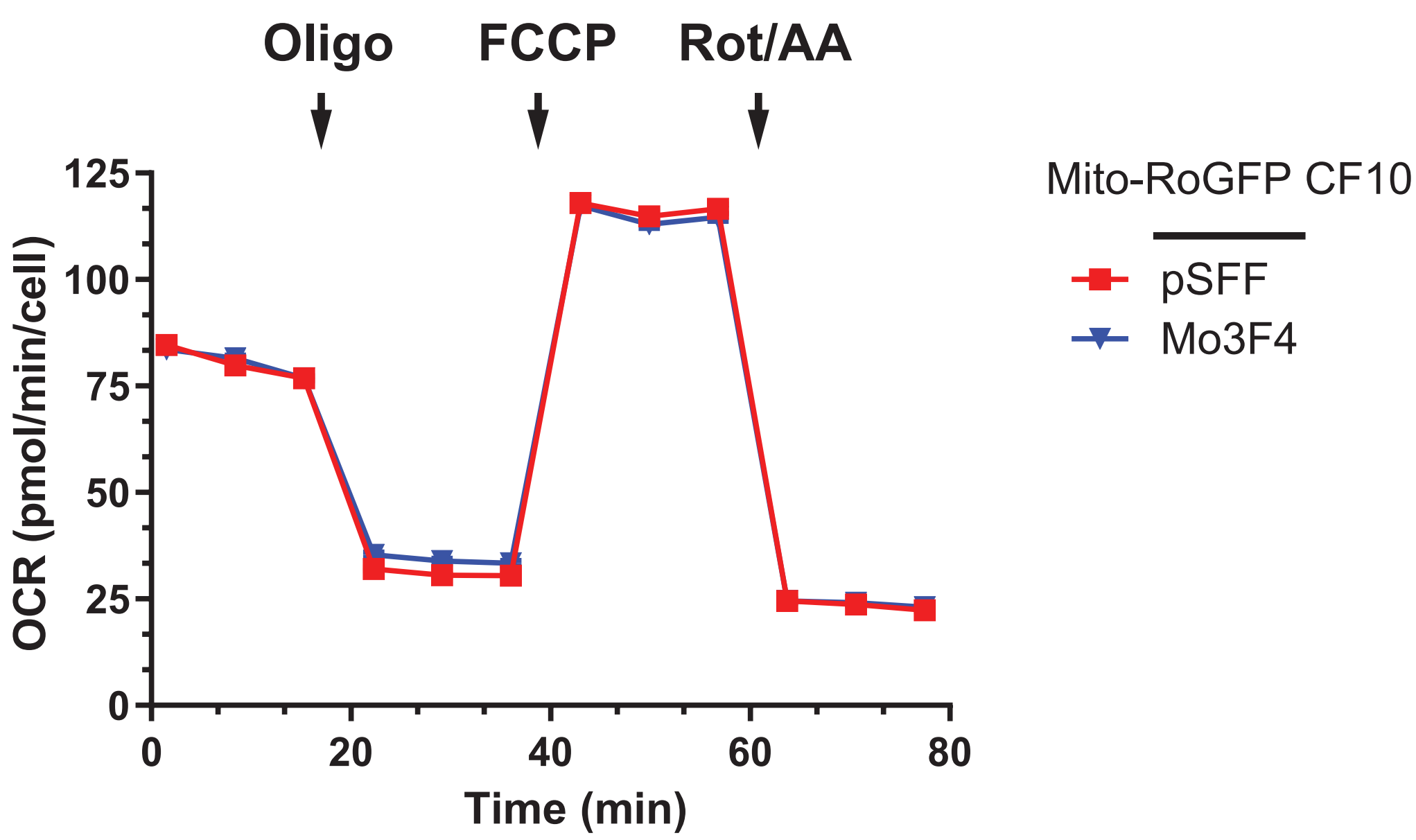

Fig. S9. Expression of PrP-Mo3F4 or vector alone does not significantly impact mitochondrial respiration in Mito-RoGFP CF10 cells. Mitochondrial respiration in Mito-RoGFP CF10-Mo3F4 cells or Mito-RoGFP cells expressing the pSFF vector alone (Mito-RoGFP CF10-pSFF) was measured using the Seahorse XFe96 Analyzer. Oxygen consumption rate (OCR) was measured in picomoles (pmol) per minute (min)/cell. Cells were treated with oligomycin (oligo), FCCP, and rotenone/antimycin A (Rot/AA) to determine mitochondrial activity. The OCR was normalized to cell number and all plots show the average OCR in pmol/min/cell ± SEM for n=16 replicates.

Journal of Cell Science • Supplementary information

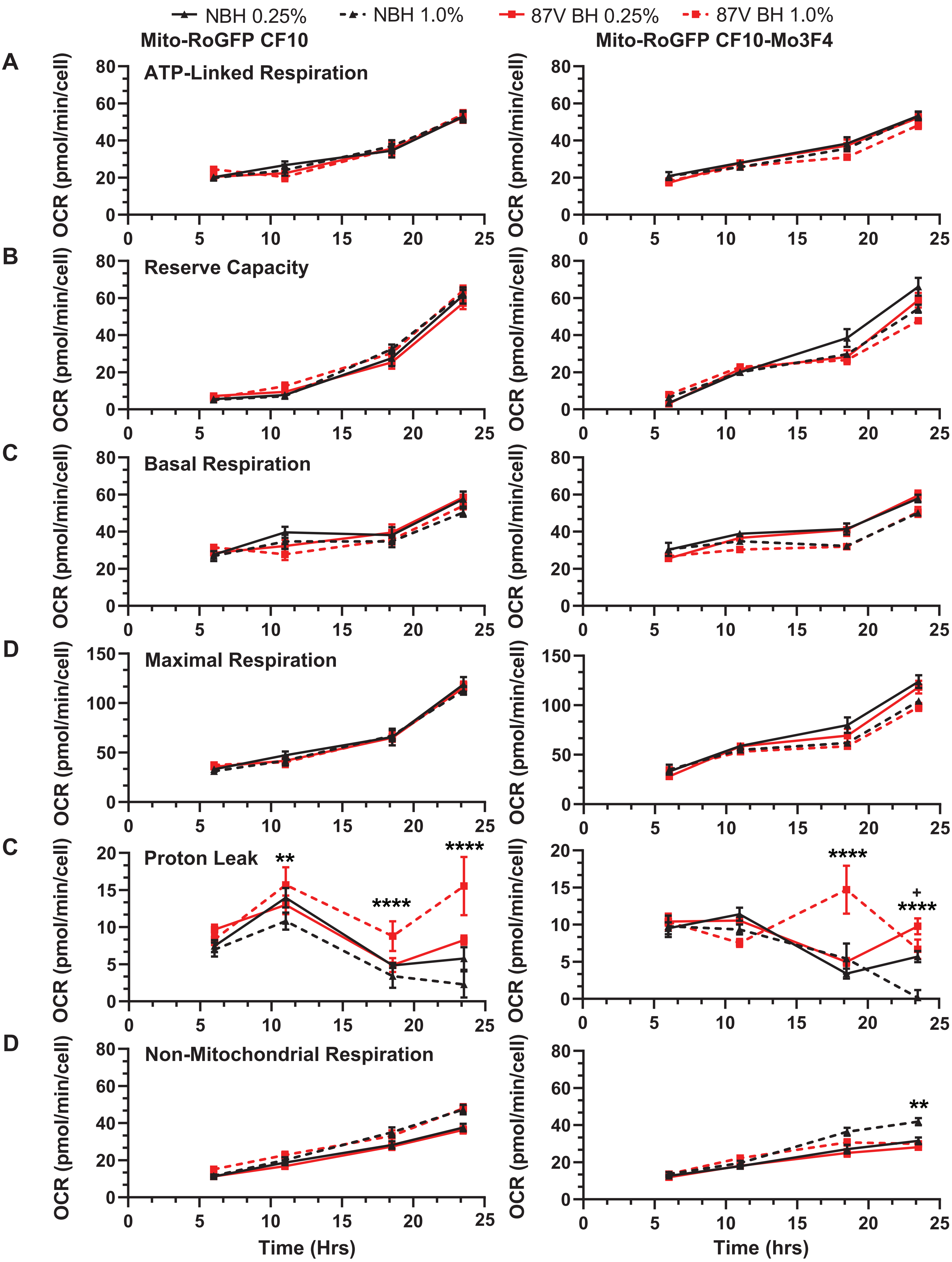

NBH 0.25%
NBH 1.0%
87V BH 0.25%
87V BH 1.0%
Mito-RoGFP CF10
Mito-RoGFP CF10-Mo3F4
A
ATP-Linked Respiration
B
Reserve Capacity
C
Basal Respiration
D
Maximal Respiration
C
Proton Leak
D
Non-Mitochondrial Respiration
OCR (pmol/min/cell)
Time (Hrs)
Time (hrs)
**
****
****
****
+
****
**

Fig. S10. No effect of higher concentrations of 87V BH on mitochondrial respiration. Mitochondrial oxygen consumption rate (OCR) as determined using the Seahorse XFe96 Analyzer for CF10 (left panels) and CF10-Mo3F4 (right panels) cells treated with oligomycin, FCCP, and rotenone/antimycin A. Data were used to calculate mitochondrial respiration states (A) ATP-linked Respiration, (B) Reserve Capacity, (C) Basal Respiration, (D) Maximal Respiration, (E) Proton Leak, and (F) Non-mitochondrial respiration. Cells were exposed to 0.25% (solid lines) and 1% (dashed lines) C57BL/10 Normal brain homogenate (NBH, black lines and triangles) and 87V BH (red lines and squares), and OCR in picomoles (pmol) per minute (min) per cell was assayed from 6 to 24 hrs. The OCR was normalized to cell number and all plots show the average OCR in pmol/min $\pm$ SEM for n=8 replicates. Statistical significance was calculated by two-way ANOVA using Tukey's multiple comparisons test. Plus signs and asterisks respectively indicate the differences in samples exposed to 0.25% and 1% BH. $p^{**} = 0.01 – 0.005$, $p^{****} = < 0.0009$, $p^{+} = 0.02$.

Journal of Cell Science • Supplementary information

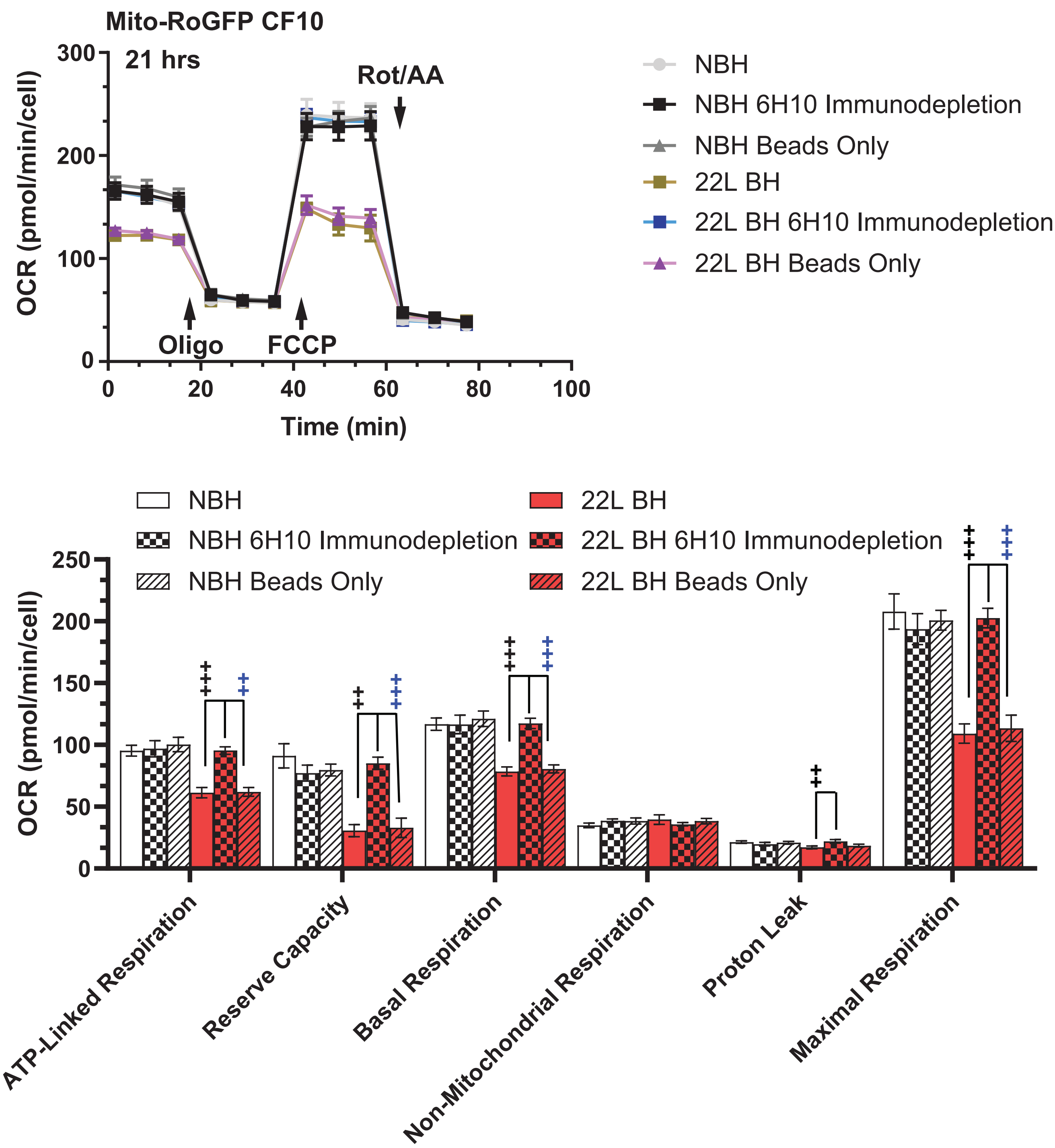

Journal of Cell Science • Supplementary information

Fig. S11. Changes in mitochondrial respiration are dependent on the presence of 22L PrP^{Sc} in the brain homogenate. Mito-RoGFP CF10 cells were exposed to C57BL/10 normal brain homogenates (NBH, light gray lines, open bars), 22L brain homogenate (22L BH, green lines, red bars) incubated with protein G agarose beads alone (Beads Only, dark gray and plum lines for NBH and 22L respectively, lined bars), or 22L BH incubated with the 6H10 antibody followed by protein G-coupled agarose beads (6H10 immunodepletion, black and blue lines for NBH and 22L respectively, checkered bars) to deplete PrP^{Sc} from the homogenate. Mitochondrial oxygen consumption rate (OCR) was determined using the Seahorse XFe96 Analyzer for CF10 (upper panel) cells treated with oligomycin (oligo), FCCP, and rotenone /antimycin A (Rot/AA). Data were used to calculate mitochondrial respiration states (lower panel) of ATP-linked Respiration, Reserve Capacity, Basal Respiration, Non-Mitochondrial Respiration, Proton Leak, and Maximal Respiration. Data shown are averages and SEM of n=8 replicates. Statistical significance was calculated by two-way ANOVA using Tukey's multiple comparisons test. Black plus signs indicate the difference between 6H10 immunodepletions and 22L BH while blue plus signs indicate the difference between 6H10 immunodepletions and beads only samples. $p^{++} = 0.01 – 0.005$, $p^{+++} = 0.005 – 0.001$.

Journal of Cell Science • Supplementary information

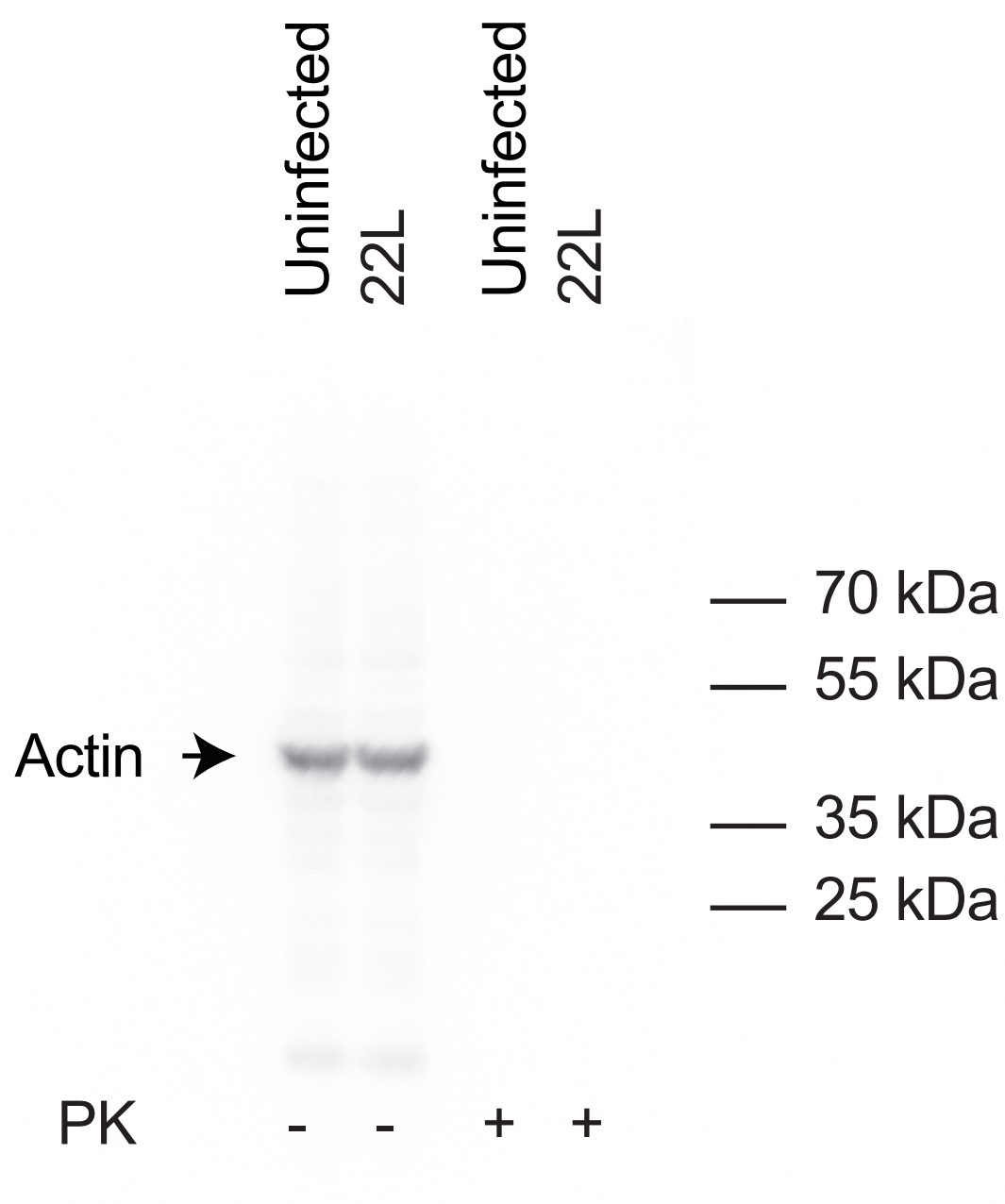

No PK Uninfected / 22L Pixel Intensity Ratio = 1.05

Fig. S12. Similar protein concentrations in CF10-Mo3F4 cell lysates prior to PK treatment. Western blot of the actin protein loading controls for the non-PK treated samples (-) for the blots shown in Figure 7. The blots show that the actin levels for CF10-Mo3F4 cell lysates were identical prior to PK treatment (+) to detect PrP^{Sc}. Actin is indicated by the arrow and molecular weight markers are shown on the right. For PK untreated samples, the ratio actin in un-infected and 22L infected samples was calculated.

Journal of Cell Science • Supplementary information

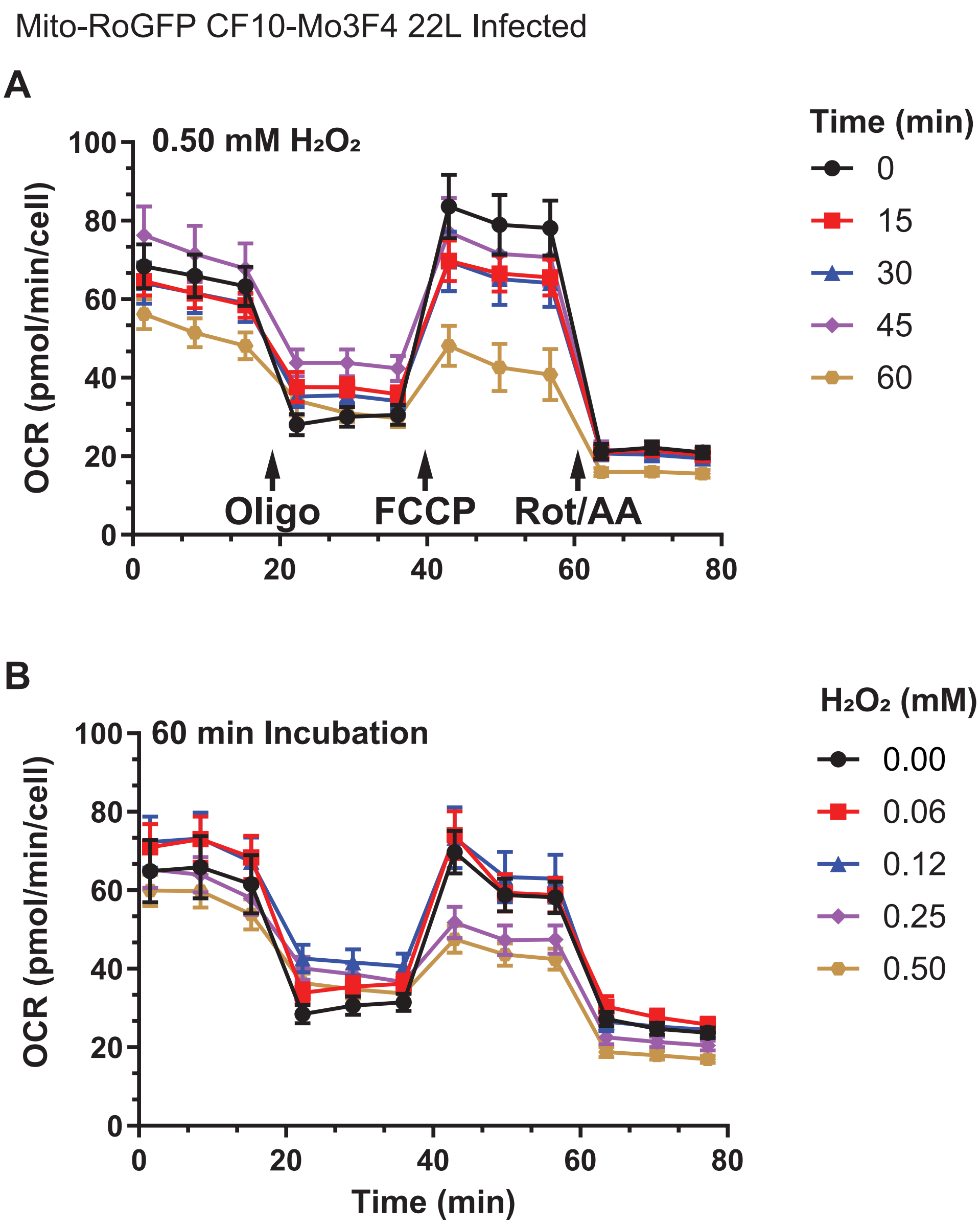

Fig. S13. Mitochondrial respiration in 22L infected CF10-Mo3F4 cells is significantly reduced following exposure to 0.5 mM hydrogen peroxide for 60 min. Mitochondrial respiration was recorded for 22L infected CF10-Mo3F4 cells after exposure to (A) 0.5 mM hydrogen peroxide (H_2O_2) for 0 (black lines and circles), 15 (red lines and squares), 30 (blue lines and triangles), 45 (plum lines and diamonds), and 60 (gold lines and hexagons) min or treated for 60 min with (B) 0 (black lines and circles), 0.06 (red lines and squares), 0.12 (blue lines and triangles), 0.25 (plum lines and diamonds), and 0.5 (gold lines and hexagons) mM hydrogen peroxide (H_2O_2). Cells were treated with oligomycin (oligo), FCCP, and rotenone/antimycin A (Rot/AA) to assess mitochondrial function. Oxygen consumption rate (OCR) was measured in picomoles (pmol) per minute (min). The OCR was normalized to cell number and all plots show the average OCR in pmol/min/cell $\pm$ SEM for n=8 replicates.
